# Predictors of deep brain stimulation response in patients with obsessive compulsive disorder: a systematic review and meta-analysis

**DOI:** 10.1038/s41598-026-54929-8

**Published:** 2026-06-04

**Authors:** Santhosh G. Thavarajasingam, Sajeenth Vishnu K., Amir Puyan Divanbeighi Zand, Daniele S. C. Ramsay, Ahmed Salih, Manuel V. Baby, Roma D. Thakker, Jasleen Nagi, John Eraifej, Guru Amirthalingam, Zoe Shaked, Hugo Sivov, Dragan Jankovic, Andreas Kramer, Denise Linsmayer, Andreas Nowacki, Sergiu Groppa, Martin B. Glaser, Jan Hinnerk-Mehrkens, Florian Ringel, Alexander L. Green

**Affiliations:** 1 Oxford Functional Neurosurgery, John Radcliffe Hospital, Oxford, UK; 2 Nuffield Department of Clinical Neurosciences, University of Oxford, Oxford, UK; 3 Nuffield Department of Surgical Sciences, University of Oxford, Oxford, UK; 4 Faculty of Medicine, Imperial College London, London, UK; 5 Imperial Brain and Spine Initiative, London, UK; 6 Department of Neurosurgery, Charité – Universitätsmedizin Berlin, Corporate Member of Freie Universität Berlin and Humboldt-Universität zu Berlin, Berlin, Germany; 7 Department of Neurosurgery, Inselspital – University Hospital of Bern, Bern, Switzerland; 8 Department of Psychiatry, Vitos Klinikum Rheingau, Eltville am Rhein, Germany; 9 Department of Neurology, University Medical Center Mainz, Mainz, Germany; 10 Department of Neurosurgery, University Medical Center Mainz, Langenbeckstraße 1, 55131 Mainz, Germany; 11 Department of Neurosurgery, LMU University Hospital, LMU Munich, Munich, Germany

**Keywords:** Obsessive-compulsive disorder, OCD, Deep brain stimulation, DBS, Predict, Predictor, Responder, Response, Responsiveness, Outcome, Obsessive compulsive disorder, Translational research, Neuroscience, Medical research

## Abstract

Obsessive-compulsive disorder (OCD) is a chronic and debilitating condition, often resistant to conventional treatments. Deep brain stimulation (DBS) emerges as a promising intervention, but its efficacy varies among patients, underscoring the need to understand the predictive factors influencing its outcomes. To evaluate and compare disease and patient characteristics in their ability to predict response to DBS in OCD patients. All major databases were searched for original studies. This study differentiated responders, partial responders, and non-responders based on relative post-treatment Y-BOCS score changes and pre-defined Y-BOCS cut-off scores. Clinical predictors were assessed using qualitative synthesis, univariate analysis, stepwise and regularisation-tuned multivariate linear and logistic regression analyses. The meta-analysis, comprising 28 studies with a pooled sample of 296 patients, found that higher baseline Y-BOCS scores significantly predicted favourable long-term DBS response in the multivariate regression analysis (*p* = 0.0075), whereas antipsychotic use was identified as a significant predictor of non-response (*p* = 0.0138). In the univariate analysis, antidepressant use was negatively associated with DBS response (*p* = 0.027), while anxiolytic use was positively associated with short-term improvement (*p* = 0.025). Symmetry, hoarding, and perfectionism symptomology at baseline predicted reduced short-term improvement (*p* = 0.027). Adjusting for stimulation target, aggression, and intrusive thoughts, baseline symptomology emerged as a positive predictor of DBS response in the multivariate model (*p* = 0.0475). In the multivariate analysis, excluding studies with high risk of bias, the symptom combination anxiety, avoidance, and fear was associated with DBS response (*p* = 0.024). Disease duration, gender, anatomical target location, and age did not predict DBS response (*p* > 0.05). This study found that a higher baseline severity of OCD symptoms significantly predicted a greater likelihood of long-term response to DBS. In contrast, the use of antipsychotics and antidepressants was associated with poorer outcomes, while anxiolytic use appeared to support short-term improvement. Compulsive symptom profiles characterised by symmetry, hoarding, and perfectionism were associated with non-response, whereas the presence of aggression and intrusive thoughts, as well as anxiety, fear and avoidance symptomology, was linked to a positive response. Further research with homogenous methodology and outcome reporting, as well as randomised control trials, are required to further elucidate these phenotypes and allow for more personalised and, thus, likely more effective DBS treatment strategies for all OCD patients.

**Supplementary Information:**

The online version contains supplementary material available at 10.1038/s41598-026-54929-8.

## Introduction

Obsessive-compulsive disorder (OCD) is a severe and common psychiatric illness, with a lifetime prevalence ranging between 1–3%^1^. OCD is characterised by intrusive and recurrent thoughts (obsessions) and/or repetitive behaviours (compulsions) in response to obsessions. These ego-dystonic behaviours frequently give rise to distress. Psychological therapy, namely exposure-based response prevention (ERP) and cognitive behavioural therapy (CBT), is the first line in targeting OCD-related functional impairments^1^. Such behavioural therapy has demonstrated a greater remission rate of OCD than 2nd line pharmacotherapy (57% and 47%, respectively)^2^. Recommended pharmacotherapy regimens include selective serotonin reuptake inhibitors (SSRIs) and clomipramine^1^. Nevertheless, 10% of OCD patients will remain treatment refractory despite receiving maximal combined psycho-pharmacotherapy^3^. In such severe, treatment-refractory cases of OCD, deep brain stimulation (DBS) has demonstrated significant efficacy in improving outcomes.

Deep brain stimulation is an established treatment for severe, treatment-refractory OCD, with over two decades of clinical application, although access and implementation remain limited in many healthcare systems; although randomised controlled trial (RCT) evidence now exists for efficacy, with calls for integration into routine clinical practice^4^. It has frequently faced hesitancy from clinicians in the past, given its invasive nature and the well-recognised stigma of utilising neurosurgery to treat psychiatric illness^5^. Yet, DBS is garnering growing interest as an evidence-based therapy. Especially DBS of the ventral anterior limb of the internal capsule (vALIC) and the ventral capsule/ventral striatum (VC/VS)^6^.

Though DBS therapy has elicited promising symptomatic benefits, 2021 NICE recommendations concluded that the evidence surrounding safety and efficacy was inadequate to be included in their OCD treatment recommendations^7^. Recent evidence suggests that the DBS connectivity profile is a significant predictor of treatment response, however, there is a lack of clarity surrounding preoperative clinical prognostic indicators^8^. This systematic review and meta-analysis evaluates clinical predictors of DBS response in OCD patients, leveraging data from the latest randomised control trials, cohort studies, and case series.

## Methods

### Search strategy and selection criteria

**Fig. 1:**
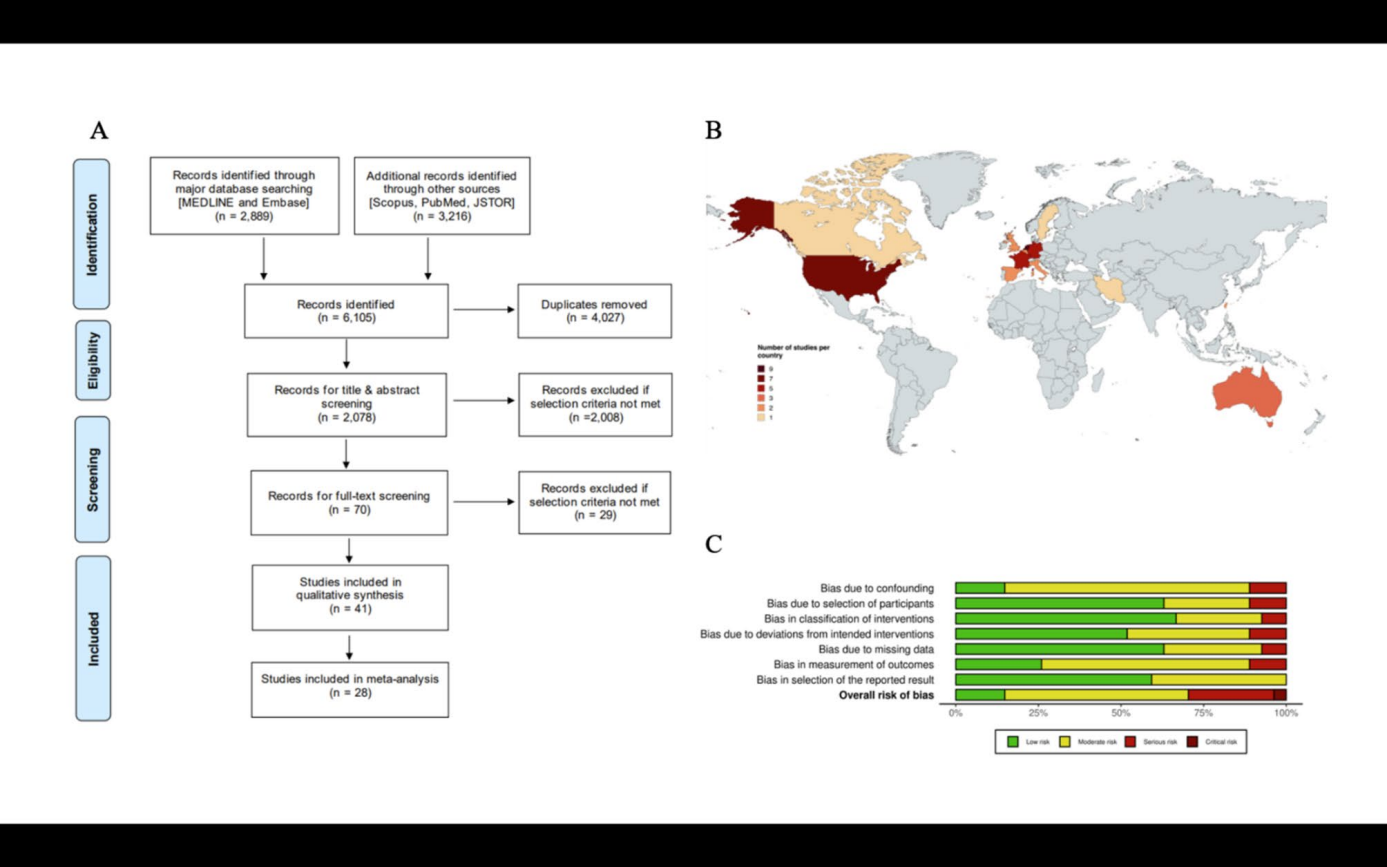
**A** presents a Preferred Reporting Items for Systematic Reviews and Meta-Analyses (PRISMA) flowchart, which delineates the systematic selection and screening procedure for the studies included in our meta-analysis. The flowchart’s stepwise structure encapsulates the progressive narrowing down of studies based on the predefined inclusion and exclusion criteria, ultimately leading to the final selection of articles included in this review. **B** illustrates a colour-coded world map, representing the geographical distribution and density of studies (*n* = 41) included in the systematic review. Countries contributing to international multi-centre trials (*n* = 35) are depicted individually. The colour gradient underscores the scientific contributions across regions, with each hue corresponding to the number of studies. The Netherlands has the highest representation (*n* = 9), followed by the United States (*n* = 7), Germany (*n* = 5), France (*n* = 4), and Australia (*n* = 3). Other countries such as Spain, Italy, the United Kingdom, Belgium, and Taiwan contributed two studies each; while Canada, Sweden, and Iran are each represented by a single study. **C** exhibits a risk of bias summary plot for non-randomized studies with bar chart of the distribution of risk-of-bias judgments for all included studies (*n* = 31) across the domains of the ROBINS-I tool, shown in percentages (%) is shown. In the bottom, an overall risk of bias, which represents the collated risk-of bias judgements for all domains, is depicted.

This systematic review was registered on PROSPERO CRD42022338915 under the title “Predictors of response to deep brain stimulation (DBS) in the management of obsessive-compulsive disorder (OCD)” on the 14th of June 2022. It was conducted using the guidelines outlined by the Cochrane Collaboration and the Preferred Reporting Items for Systematic Reviews and Meta-Analyses (PRISMA)^9^. The completed PRISMA flowchart is shown in Fig. 1A. The PRISMA 27-point checklist can be found in Supplementary Table 1. The literature search was carried out on the 22nd of June 2022, using a search of MEDLINE, Embase, Scopus, PubMed, and JSTOR from 1943 to 2022. The complete search strategy can be found in Supplementary Table 2.

The inclusion and exclusion criteria for both the systematic review and meta-analysis are detailed in Supplementary Table 3. Only original studies reporting preoperative patient and disease characteristics, as well as other clinical predictive variables in responders and non-responders to OCD DBS treatment, were included. Studies comparing responders (defined primarily as a ≥ 35% reduction in Yale–Brown Obsessive–Compulsive Scale [Y-BOCS] score following DBS) to non-responders were eligible. Minor study-level variations in response definitions were accepted where explicitly reported. For the meta-analysis, only studies providing comparative aggregated patient-level data—specifically, comparisons of patient and disease characteristics between DBS responders and DBS non-responders (and/or partial responders), or studies offering comparative data on at least one of these response subgroups—were included. Case reports with single-patient cases, as well as reviews, were excluded.

All included papers were assessed for eligibility by two independent reviewers (SGT & APDZ). Any disagreements were resolved by consensus after discussion with a third reviewer (DSCR). All included papers were assessed for eligibility by two independent reviewers (SGT & APDZ). Any disagreements were resolved by consensus after discussion with a third reviewer (DSCR).

### Data analysis

All relevant data were extracted manually using the Covidence data collection tool^10^. A list of extracted variables can be found in Supplementary Table 4. Data from each study were extracted by a single reviewer (SGT, APDZ, DSCR, SVK, RDT, HS, SA, ZS). All relevant data were extracted manually using a predefined extraction template. The first and second authors (SGT and APDZ) independently cross-checked all extracted data in full, blinded to each other’s assessments, and any discrepancies were resolved through discussion to ensure consistency and accuracy. In case of missing data, the respective study’s corresponding author was contacted. All articles were critically appraised, and the risk of bias was determined against all the domains of either the ROBINS-I (non-randomised studies; 2016 version)^11^ or the RoB-2 tool (randomised studies; 2019 version)^12^ by two independent reviewers 2019 version) by two independent reviewers (SVK & DSCR), and a consensus was reached by discussion with a third reviewer (ST). The results of the ROBINS-I can be found in Fig. 1C and Supplementary Fig. 1, with RoB-2 analyses in Supplementary Fig. 2. Furthermore, the level of evidence for each included article was scored using the 2011 version of the Oxford Centre for Evidence-Based Medicine (CEBM) Levels of Evidence Table (Supplementary Table 5)^13^ by two independent reviewers (SVK & ZS), and a consensus was reached by discussion with a third reviewer (RT). Definitions of DBS response used by each study are shown in Table 1.

**Table 1:** Study characteristics of the included studies in this systematic review (*n* = 41).

Study (Year)	Study design	Sample (Male)	DBS methodology	DBS target site	Imaging modality	Definition of FR (Definition of PR)	Number of responders (%)	Y-BCOS follow up; Short term (ST)/Long term (LT)	Follow-up period
Abelson et al. (2005)	Prospective Double-blind randomised crossover trial	4 (2)	MRI guided stereotactic placement of quadripolar stimulating electrodes. Stimulation based on exploratory testing with parameters titrated based on acute effects within 3–8 days	ALIC	MRI, PET	≥ 35% decline in Y-BOCS (NR)	1 (25)	ST + LT	13 months
Acevedo et al. (2023)	Cohort	8 (5)	MRI guided stereotactic placement of quadripolar stimulating electrodes. Initial optimisation phase in which CBT is commenced following a 6-point reduction in Y-BOCS after stimulation, followed by a maintenance phase of ongoing psychopharmacotherapy.	NAcc	MRI, CT	≥ 35% decline in Y-BOCS	6 (75)	ST + LT	47 months
Baldermann et al. (2019)	Cohort	22 (9)	Implantation of 2 quadripolar DBS electrodes. Stimulation commenced with connectivity estimation phase to enable region of interest and fibre tract analysis.	ALIC/NAcc	CT, MRI	NR	NR	ST	12 months
Barcia et al. (2019)	Double-blind randomised trial	7 (3)	CT/MRI guided electrode placement. Randomised sequenced contact activations (C0,1,2,3 and sham) were generated for each participant with 3-month stimulation, 1 month washout period.	NAcc	CT, MRI	≥ 35% decline in Y-BOCS (NR)	6 (85.71)	ST + LT	19 months
Chabardes et al. (2020)	Cohort	19 (7)	Implantation with post-operative MRI/CT confirmation and trials of monopolar stimulation- 6-month chronic phase stimulation monitored with 0.5 V step increases until side effects.	Non-motor area of STN	CT, MRI	≥ 35% decline in Y-BOCS (NR)	14 (73.68)	ST + LT	24 months
Denys et al. (2010)	Double-blind randomised crossover trial	16 (9)	MRI guided quadripolar electrode placement. Open phase of 8 months when parameters were optimised through voltage changes (maximum 5 V)	NAcc	CT, MRI	≥ 35% decline in Y-BOCS (NR)	9 (56.25)	ST + LT	21 months
Denys et al. (2020)/(Graat et al. (2021)	Cohort	70 (22	Stereotactic guided implantation of electrodes in the vALIC. Stimulation initiated after two weeks to optimise parameters before CBT is started.	vALIC	MRI	≥ 35% decline in Y-BOCS (≥ 25 decline in Y-BOCS)	36 (51.4)	ST	12 months
Farrand et al. (2018)	Case-series	7 (3)	2 Stage DBS surgery, utilising IPG. 8-month stimulation commenced 2-weeks post-operatively, with titration.	NAcc, BNST	EEG, MRI, SPECT	≥ 35% decline in Y-BOCS (≥ 15% decline in Y-BOCS)	3 (42.95)	LT	31 months
Germann et al. (2022)/Lee et al. (2019)	Cohort	5 (2)	MRI guided DBS electrode insertion. Connectivity analyses with stimulation maps utilised.	ALIC, Inferior thalamic peduncle	CT, MRI, PET	≥ 35% decline in Y-BOCS (NR)	5 (100)	LT	24 months
Graat et al. (2021)	Cohort	50 (16)	Implantation of electrodes bilaterally targeted at the vALIC, as well as an implantable pulse generator (IPG). Stimulation was initiated in an optimisation phase two weeks after implantation, and once Y-BOCS scores decreased > = 6 points and symptoms stopped reducing further, CBT was started.	vALIC	NR	≥ 35% decline in Y-BOCS (≥ 25 decline in Y-BOCS)	25 (50)	LT	82 months
Graat et al. (2022)	Case-series	6 (2)	Stereotactic surgery for implantation of quadripolar DBS electrodes. Stimulation commenced 2-weeks post operatively with DBS settings optimised.	vALIC (medial forebrain bundle)	MRI	≥ 35% decline in Y-BOCS (≥ 25 decline in Y-BOCS)	4 (66.7), 1 (16.7) partial	LT	98 months
Greenberg et al. (2006)	Cohort	10 (6)	Stereotactic surgery for implantation of quadripolar electrodes under MRI guidance. Stimulation period involved Intra-operative test stimulation, 3-week monopolar survey followed by chronic DBS stimulation.	ALIC, VC/VS	CT, MRI	≥ 35% decline in Y-BOCS (≥ 25% decline in Y-BOCS)	4 (50)	ST + LT	36 months
Hartmann et al. (2016)	Cohort	6 (3)	Tractography- Activation Models for analysis of axonal activation by specific DBS per patient.	ALIC, NAcc	CT, MRI	≥ 50% decline in Y-BOCS (≥ 10decline in Y-BOCS)	4 (67)	LT	24 months
Holland et al. (2020)	Cohort	9 (5)	2 stage DBS surgery with IPG implantation. Stimulation commenced 2–4 weeks post-implantation with voltage adjustments made.	ALIC, VC/VS	CT, MRI	≥ 35% decline in Y-BOCS (NR)	6 (66.7)	LT	54.8 months
Huff et al. (2010)	Double-blind randomised crossover trial	10 (6)	6-month stimulation; 3-month DBS, followed by 3-month sham stimulation	Unilateral right NAcc	CT, MRI	≥ 35% decline in Y-BOCS (≥ 25% decline in Y-BOCS)	1 (10) full, 5 (50) partial	ST + LT	12 months
Huys et al. (2019)	Cohort	20 (10)	Quadripolar bilateral lead implantation under MRI and stereotactic CT, connected to an IPG. Mean 12-month stimulation- final stimulation setting set 1–2 weeks after implantation. Settings were continuously optimised.	ALIC/NAcc	CT, MRI	≥ 35% decline in Y-BOCS (≥ 25% decline in Y-BCOS)	8 (40) full responders; 6 (30) partial responders	ST + LT	12 months
Islam et al. (2015)	Case-series	8 (7)	Stimulation parameters determined after electrode and pulse generator implantation	BNST/NAcc	T1, T2 & Fluid attenuated inversion MRI; CT	≥ 35% decline in Y-BOCS (NR)	6 (75)	ST + LT	40.8 months
Kahn et al. (2021)	Case-series	5 (4)	Implantation of 4 bilateral contact electrodes. Initial and ongoing programming post-implantation for optimisation.	ALIC/AC	CT, MRI	NR	NR	ST + LT	12–48 months
Li et al. (2020)	Cohort	50 (24)	Connectivity analysis following stimulation of 4 different regions.	ALIC/STN/NAcc/ALIC + STN	CT, MRI	NR	NR	ST	ALIC DBS Cohort = 12 months; STN DBS Cohort = 12 months; NAcc DBS Cohort = 3 months; ALIC + STN DBS cohort = NR
Liebrand et al. (2019)	Cohort	12 (2)	Stereotactically planned implantation of quadripolar electrodes. DBS activation 2-weeks post-implantation with 6-month average optimisation and adjustment phase, followed by stable stimulation phase	B vs. ATR specific targeting	CT, DWI, MRI, PET	≥ 35% decline in Y-BCOS (NR)	7 (58)	LT	12 months
Liebrand et al. (2021)	Cohort	57 (16)	Stereotactic implantation of electrodes. Average 12-month stimulation; DBS activation 2-weeks post-implantation followed by optimisation and stimulation phase.	NAcc	CT, MRI	≥ 35% decline in Y-BOCS (NR)	31 (54.3)	LT	12 months
Lopez-Sosa et al. (2021)	Cohort	9 (5)	On/Off stimulation states completing task-blocks, with intermediary 5-minute ‘wash out’ period	NAcc	CT, EEG, MRI	NR	NR	LT	33 months
Luyten et al. (2016)	Double-blind randomised crossover trial	24 (12)	Bilateral stereotactic implantation of quadripolar electrodes. Initial parameter optimisation followed by either ON or OFF stimulation phases. Following completion of cross-over arms was optional stimulation phase.	BNST, IC, ALIC	MRI	≥ 35% decline in Y-BOCS (NR)	16 (67)	LT	77 months
Mallet et al. (2008/2019)	Double-blind randomised crossover trial	16 (10)	Stereotactic MRI and ventriculography-guided electrode implantation. Half the cohort underwent active stimulation for three months, followed by a 1-month washout period and subsequent crossover to sham stimulation.	STN	MRI	> 35% reduction in Y-BOCS	9 (56.3)	LT	46 months
Mosley et al. (2021)	Double-blind randomised crossover trial	9 (5)	Bilateral implantation of quadripolar electrodes connected to IPG. One-month recovery phase post-op, 3-month double-blind phase followed by open label trial with stimulation on.	NAcc (BNST)	CT, MRI	≥ 35% decline in Y-BOCS (NR)	7 (77.7)	LT	12 months
Naesstrom et al. (2021)	Cohort	11 (4)	MRI guided electrode implantation. Average 12-month duration- DBS commenced within 1-month post-operatively, 3-6month titration phase for optimisation.	BNST	CT, MRI	≥ 35% decline in Y-BOCS (≥ 25% decline in Y-BCOS)	6 full (54.5), 4 partial (36.3)	LT	12 months
Ooms et al. (2014)	Cohort	16 (7)	Bilateral implantation of DBS electrodes. 8-month active first phase stimulation (T1), followed by 3-5years of stimulation (second phase T2)	NAcc	NR	≥ 35% decline in Y-BOCS (NR)	11 (68.7)	LT	36 to 60 months
Parvaresh-Rizi et al. (2022)	Case-series	4 (0)	Stereotactic implantation of electrodes under MRI guidance. Stimulation commenced 5-days post-operatively at set parameters, followed by optimisation phase.	vALIC/NAcc	MRI	≥ 35% decline in Y-BOCS (NR)	0 (0), statistically none achieved > 35% decline, however 2 patients reported complete remission of symptoms	ST + LT	12 months
Raymaekers et al. (2017)	Cohort	24 (12)	Varied time periods of stimulation status (ON/OFF) analysed via linear mixed model against outcome measures in DBS patients.	IC/BNST	NR	≥ 35% decline in Y-BOCS (NR)	67%	ST + LT	76.5 months
Sildatke et al. (2021)	Cohort	15 (6)	Bilateral implantation of electrodes. 6-12month ongoing stimulation with DBS off-states (12–24 h); variable amongst participants	ALIC-NAcc	EEG, MRI, CT	ΔMFT and ERN with *p* < 0.05 considered significant	NR	LT	7.6 months
Tsai et al. (2012)	Case-series	4 (4)	Implantation of quadripolar electrodes. Post-operative test stimulation 2-weeks post-implantation. Fixed stimulation settings across chronic treatment phase.	VC/VS	MRI, CT	Stimulation-induced smile/laughter (NR)	NR	LT	15 months
Tsai et al. (2014)	Case-series	4 (4)	Post-operative test stimulation 2-weeks post-implantation. Initially fixed stimulation settings with parameters adjusted for optimisation.	VC/VS	FDG-PET	Mean change of Y-BOCS score, *p* < 0.05 considered significant	100%	LT	15 months
Tyagi et al. (2019)	Double-blind randomised trial	6 (5)	Stereotactic 1.5 MRI utilised for implantation. Stimulation commenced 4-weeks post-implantation. 6 12-week stimulation phases followed; (1) double-blind stimulation of the 2 target sites (2) stimulation of alternate site (3) open phase combined stimulation (4) and (5) open phases for optimised stimulation (6) simultaneous CBT with stimulation.	VC/VS, amSTN	MRI	≥ 35% reduction in Y-BOCS (NR)	amSTN phase − 3/6 (50), VC/VS phase − 5 of 6 (83) COMB phase − 5 of 6 (83) OPT phase − 6/6 (100) OPT plus adjunctive CBT phase − 6/6 (100)	ST + LT	6 months
vanderVlis et al. (2021)	Cohort	8 (2)	Implantation of bilateral quadripolar electrodes, connected to an IPG. Average 26-month duration- stimulation parameters regularly optimised.	VC/VS	MRI, CT	≥ 35% reduction in Y-BOCS (NR)	5 (63)	LT	26.25 months
Voon et al. (2018)	Double-blind randomised trial	12 (4)	Task performing assessment across two randomised arms- alternating between ON and OFF stimulation days.	STN	NR	NR	NR	LT	38.1 months
Welter et al. (2011)	Cohort	12 (7)	Bilateral implantation of stimulating electrodes. 3-month stimulation post-implantation and off-line analysis	STN	Pre-op MRI	NR	NR	LT	3 months
Widge et al. (2022)	Cohort	8 (NR)	Post-implantation stimulation, patient-specific tractography utilised for analysis.	VC/VS	MRI, CT scans, whole brain tractography	≥ 35% reduction in Y-BOCS (NR)	5 (63) Overall responder: 3/8 (38)	LT	24–60 months
Winter et al. (2021)	Case-series	6 (4)	Bilateral implantation of electrodes. Stimulation parameters adjustment and titration with post-operative testing.	BNST/ALIC	MRI, CT	≥ 40% reduction in Y-BOCS) (NR)	4/6 − 2 remitted (66.7)	ST + LT	48–98 months

A total of 41 studies were included in this systematic literature review (SLR), with six publications counted as three pairs of studies (namely Denys 2020 & Graat 2021, Germann 2022 & Lee 2019, Mallet 2008 & 2019). For accuracy, these studies were treated as separate entries in the PRISMA flow diagram and in-text citations; however, since they reported findings from the same patient samples, their results have been combined in the summary tables. Key variables including study design, sample size along with the gender composition, DBS methodology, imaging modalities utilised, the definition of treatment response, number of responders, and the follow-up duration have been systematically tabulated for each study. MFT refers to conflict-related medial frontal theta, a brainwave pattern from the medial frontal cortex (MFC) detected by EEG that is associated with focussed attention and cognitive control. Error-related negativity (ERN) is an EEG pattern generated in the posterior MFC. Significant presence of these patterns (p < 0.05) was used by one study (Sildatke et al.) to define responders to DBS. The abbreviations used in the table are as follows: Anterior Limb of Internal Capsule (ALIC), Anterior Thalamic Radiation (ATR), Bed Nucleus of Stria Terminalis (BNST), Computed tomography (CT), Deep Brain Stimulation (DBS), Error-related Negativity (ERN), Full response (FR), Implantable Pulse Generator (IPG), Inferior Thalamic Peduncle (ITP), Magnetic Resonance Imaging (MRI); Medial Frontal Theta (MFT), Mid Forebrain Bundle (MFB), No data Reported (NR), Nucleus Accumbens (NAcc), Partial response (PR), Subthalamic Nucleus (STN), Ventral Anterior Limb of Internal Capsule (vALIC), Ventral Capsule (VC), Ventral Striatum (VS), Yale-Brown Obsessive Compulsive Score (Y-BCOS).

Statistical analysis and plot synthesis were carried out using the meta package with R software (version 4.0.4) and Python (version 3.11.3)^14,15^. A dataset was additionally constructed employing Predictive Mean Matching (PMM). Chi-squared tests were applied to examine categorical clinical predictors across predefined response categories (responder, partial responder, non-responder). The statistical significance threshold was set at *p* < 0.05. Continuous clinical predictors were scrutinised using ANOVA tests, with those meeting the significance benchmark further subjected to Tukey’s HSD post-hoc tests. Univariate and stepwise multivariate regressions were performed on both the imputed and non-imputed datasets. For the final multivariate model, regularisation was employed, utilising Ridge, LASSO, and Elastic Net regression techniques. The optimal set of covariates was selected based on their performance across these regularisation methods, ensuring that the final model incorporated variables that contributed to the robustness and generalisability of the predictions. These covariates were then considered alongside findings from the univariate and stepwise multivariate regression analyses of both the unimputed and PMM-imputed datasets, as well as systematic review findings and correlation analysis results. Mixed-effect multivariate linear regressions and logistic regressions were then applied to continuous and categorical outcome variables, respectively, to yield the most important significant predictors of DBS response. A detailed account of the statistical analysis performed can be found in Supplementary Material 1.

## Results

A total of 6,105 studies were identified, of which 2,078 were abstract screened. From these, 69 full texts were assessed using our inclusion criteria. A total of 41 studies were included in this systematic review^16–56^. From these, 28 studies which fit our meta-analysis inclusion criteria were also included in the meta-analysis^17–22,24,25,27–30,32,34,36–40,44,46,47,49–52,55,56^(Fig. 1A). The total pooled sample size of the systematic review was 589, and the overall pooled sample size of the meta-analysis was 296 patients. A world map of publication origins is shown in Fig. 1B.

Out of the 41 included studies, there were 10 randomised controlled trials^46–55^and 31 non-randomised studies^16–45^. Using the ROBINS-I tool for non-randomised studies (*n* = 31), 7 were considered to have a ‘low’ risk of bias^18,23,39–43^ 16 ‘moderate’ risk of bias,^16,19–21,25,27,28,30–32,34–38,45^ 7 ‘serious’ risk of bias^17,22,24,26,29,33,44^ and 1 ‘critical’ risk of bias^56^(Fig. 1C, Supplementary Fig. 1). Using the RoB-2 tool for randomised controlled trials (n = 10), 5 were considered to have a ‘low’ risk of bias^47–49,51,52^ whereas 3 were deemed to have ‘some concerns’^46,50,53^ and 2 to have ’high concerns’^54,55^ (Supplementary Fig. 2). The OCEBM guidance was used to determine the level of evidence of each study. 15 studies were classified as level 2, 15 studies as level 3, and 11 studies as level 4 (Supplementary Table 5). The study characteristics of the included studies are detailed in Table 1, the disease and patient characteristics of the included sample in Tables 2, and the complications of DBS among the included studies in Table 3.

**Table 2:** A detailed summary of patient and disease characteristics of the DBS-treated OCD patients from the included studies (*n* = 35).

Name (year)	Predominant OCD symptoms	Mean pre-intervention Y-BOCS (SD)	Mean post-intervention Y-BOCS (SD)	Mean percentage Y-BOCS improvement (SD, *p*-value)	Number of responders (%)
Abelson et al. (2005)	Contamination (1), Intrusive images (1), Repeating (2)	32.75 (5.068)	23 (11.113)	30.25 (30.086, p-value NR)	1 (25)
Acevedo et al. (2023)	Checking (4), Washing (3), Ordering (2), Contamination (2), Self-harm (2)	30.7 (2.5)	17 (6.4)	45% (SD NR, p-value NR)	6 (75%)
Baldermann et al. (2019)	NR	31.3 (4.3)	NR	30.4% (20.1, *p* < 0.001)	NR
Barcia et al. (2019)	Contamination (3) Doubts (3), Symmetry (1)	32.28 (4.682)	NR	51.33 (21.002, p-value NR)	6 (85.71)
Chabardes et al. (2020)	Aggressive (2), Contamination (6), Doubts (11)	33.31 (3.54)	15.83 (9.61)	53.45 (27.469, *p* < 0.0001)	14 (73.68)
Denys et al. (2010)	Checking (6), Contamination (8), Symmetry (2)	33.7 (3.6)	16.2 (8.6)	52% (SD NR, *p* < 0.001)	9 (56.25)
Denys et al. (2020)/Graat et al. (2021)	Checking (13), Contamination (30), Aggressive sexual and/or religious (6)	33.7 (3.2)	NR	40% (SD NR, p-value NR)	36 (51.4%)
Farrand et al. (2018)	Contamination (3), Intrusive thoughts (3), Symmetry (1)	32.4 (3.8)	23.6 (3.7)	27.3 (15.438, *p* = 0.018)	3 (42.95)
Germann et al. (2022)/Lee et al. (2019)	NR	35 (2.098)	NR	54% (SD NR, *p* < 0.01)	5 (100)
Graat et al. (2021)	Checking (10), Contamination (21), Aggressive sexual and/or religious (3)	33.3 (3.8)	20.5 (9.9)	39% (SD NR, p-value NR)	25 (50%)
Graat et al. (2022)	Checking (2), Contamination (2), Intrusive thoughts (2)	34 (2.30)	17.8 (10.9)	48% (SD NR, *p* < 0.01)	4 (66.7), 1 (16.7) partial
Greenberg et al. (2006)	Checking (4), Contamination (1), incompleteness (3), Symmetry (1), Washing (1)	34.6 (1.685)	22.3 (SD NR)	35.5% (SD NR, *p* < 0.001)	4 (50)
Hartmann et al. (2016)	Contamination (4), Doubts (2)	NR	NR	36.2% (31.83, p-value NR)	4 (67)
Holland et al. (2020)	NR	34.2 (2.393)	20.8 (9.028)	38.75 (28.093, p-value NR)	6 (66.7)
Huff et al. (2010)	NR	32.3 (4)	25.4 (6.7)	21.7% (16.493, *p* = 0.012)	1 (10) full, 5 (50) partial
Huys et al. (2019)	NR	30.9 (4.0)	20.6 (7.4)	33.3% (21.50, *p* < 0.001)	8 (40) full responders; 6 (30) partial responders
Islam et al. (2015)	Aggressive (4), Checking (6), Compulsions (3), Contamination (3), Intrusive thoughts (3), Somatic obsession (2), Symmetry (1)	35.3 (3.33)	21 (11.8)	NR	6 (75)
Kahn et al. (2021)	Checking (1), Intrusive thoughts (1), Obsessions (5), Repetition (1), Washing (1)	35 (3)	NR	49.1 (SD NR, p-value NR)	NR
Li et al. (2020)	NR	ALIC-DBS = 31.3 (4.4); STN-DBS = 33.4 (3.7); NAcc DBS = 30 (7.75); ALIC + STN DBS = 36.2 (1.8)	ALIC-DBS = 20.7 (77); STN-DBS = 19.6 (10.6); NAcc DBS = 14.75 (7.2)	ALIC DBS Cohort = 9.6 (6.5); STN DBS cohort = 13.8 (10.8); NAcc DBS cohort = 15.1 (9.6); ALIC + STN DBS Cohort = 21.83 (5.7); p-values NR	NR
Liebrand et al. (2019)	NR	32.7 (4.3)	18.5 (6.7)	40.8% (28.1, p-value NR)	7 (58)
Liebrand et al. (2021)	NR	33.85 (3.22)	9.35 (9.40)	NR	31 (54.3)
Lopez-Sosa et al. (2021)	Checking (3), Compulsions (2), Contamination (6), Hoarding (1), Intrusive Thoughts (7), Ordering (5), Repeating (1), Symmetry (2), Washing (5)	30.8 (8.1)	NR	NR	NR
Luyten et al. (2016)	NR	34.7 (2.8)	13.2 (7.0)	60.5% (20.5, p-value NR)	16 (67)
Mallet et al. (2008/2019)	NR	30.5 (NR)	15.4 (7.0)	51.2% (21.2, p-value NR)	9 (56.3)
Mosley et al. (2021)	Aggressive (2), Checking (3), Contamination (2), Intrusive thoughts (2)	32.7 (2.6)	17.4 (SD NR)	49.6% (23.7, *p* = 0.000037)	7 (77.7)
Naesstrom et al. (2021)	Contamination (6), Religious (1), Repetitions (3), Sexual aggression (2)	33 (3.0)	20 (4.8)	@12m 38% (SD NR, *p* < 0.01)	6 full (54.5), 4 partial (36.3)
Ooms et al. (2014)	NR	33.75 (3.62)	18.00 (9.20)	NR	11 (68.7)
Parvaresh-Rizi et al. (2022)	Checking (1), Contamination (1), Intrusive thoughts (1), Obsessions (1), Symmetry (1), Washing (2)	32 (6)	26 (8)	19% (17)	2 (50)
Raymaekers et al. (2017)	NR	34.67 (NR)	NR	NR	67%
Sildatke et al. (2021)	NR	30 (5.49)	NR	12 (SD NR, p-value NR)	NR
Tsai et al. (2012)	Contamination (2), Intrusive thoughts (3), Symmetry (1)	36.3 (2.1)	24.3 (9.1)	33.06% (*P* = 0.001)	NR
Tsai et al. (2014)	Contamination (2), Intrusive thoughts (3), Symmetry (1)	36.3 (2.1)	24.3 (9.1)	33.06% (*P* = 0.001)	100%
Tyagi et al. (2019)	NR	36.17 (0.75)	amSTN 19.83 (4.32), VC/VS 17.00 (3.57), COMB 14.17 (3.18), OPT 14.33 (1.69), adCBT 9.33 (3.21)	amSTN 45.17% VC/VS 53% COMB 60.82% OPT 60.38% adCBT 74.32%	amSTN phase − 3/6 (50%), VC/VS phase − 5 of 6 (83%) COMB phase − 5 of 6 (83%) OPT phase − 6/6 (100%) OPT plus adjunctive CBT phase − 6/6 (100%)”
vanderVlis et al. (2021)	Checking (5), Contamination (4), Intrusive thoughts (3), Symmetry (3)	33.12 (3.34)	22.63 (7.91)	31.70 (SD NR, p-value NR)	5 (63)
Voon et al. (2018)	NR	34.3 (3.2)	20 (9.1)	41 (28, p-value NR)	NR
Welter et al. (2011)	NR	31.8 (3.1)	19.5 (9.5)	22 (SD NR, p-value NR)	NR
Widge et al. (2022)	NR	32.25 (3.33)	NR	46.60 (SD NR, p-value NR)	5 (63) Overall responder: 3/8 (38)
Winter et al. (2021)	Checking (3), Contamination (3), Counting (5), Intrusive thoughts (3), Ordering (2)	32.7 (5.47)	17.6 (14.1)	45.20 (SD NR, p-value NR)	4/6 − 2 remitted (66.7)

The table elucidates information on the predominant symptom dimension, the mean pre-intervention Y-BOCS score, the mean post-intervention Y-BOCS score, the mean percentage improvement in the Y-BOCS score, and the number of responders along with the percentage given in brackets. Abbreviations used within the table are defined as follows: Anterior Limb of Internal Capsule (ALIC), Cognitive Behavioural Therapy (CBT), No data Reported (NR), Subthalamic Nucleus (STN), and Yale-Brown Obsessive-Compulsive Score (Y-BOCS).

**Table 3:** A detailed summary of complications associated with DBS in OCD patient reported by all included studies.

Name (year)	Sample size (male)	Surgery-related complications (*n*, %)	Stimulation-related complications (*n*, %)	Long-term complications (*n*, %)
Abelson et al. (2005)	4 (2)	0	Tingling (1, 25.0), Throbbing (1, 25.0), Jaw sensation (1, 25.0)	0
Acevedo et al. (2023)	8 (5)	Expressive dysphasia from haematoma-associated transient lesion effect (1, 12.5)	Subcortical expressive dysphasia (1, 12.5), Generalised seizures (1, 12.5), Labile affect (1, 12.5), Fluctuating hypomania (1, 12.5)	NR
Baldermann et al. (2019)	22 (9)	NR	NR	NR
Barcia et al. (2019)	7 (3)	NR	NR	NR
Chabardes et al. (2020)	19 (7)	Contusion, headaches and hardware related (9, 47.4)	Hypomania (3, 15.8), Dyskinesia (4, 21.1), Impulsivity and disinhibition (9, 47.4), Suicide (2, 10.5)	0
Denys et al. (2010)	16 (9)	Surgical site infection (1, 6.3), Numbness at incision site (7, 43.8)	Difficulty word finding (3, 18.8), Forgetfulness (5, 31.3), Hypomania (8, 50.0), Increased libido (7, 43.8), Micturition problems (2, 12.5)	0
Denys et al. (2020)/Graat et al. (2021)	70 (22)	Pain around burr holes (12), Infection (2), Delirium (2)	Hypomanic symptoms (27), Sleeping disorder (27), Restlessness (22), Agitation (19)	0
Farrand et al. (2018)	7 (3)	0	Hypomania (2, 28.8), Déjà-vu (1, 14.3)	0
Germann et al. (2022)/Lee et al. (2019)	5 (2)	0	Removal of electrodes due to obsession related to constant stimulation (1, 20.0)	0
Graat et al. (2021)	50 (16)	Pain around wounds (8), Pain around bore holes (7), Diplopia (2)	Hypomanic symptoms (18), Restlessness (16), Fatigue (16), Impulsivity (14), Concentrating difficulty (14), Disinhibition (11), Sleep disorder (10),	0
Graat et al. (2022)	6 (2)	Device infection (1, 16.7)	Forgetfulness (1, 16.7), Headache (1, 16.7), Hypomania (1, 16.7), Restlessness (3, 50.0)	0
Greenberg et al. (2006)	10 (6)	Intracerebral haemorrhage (1, 10.0), tonic-clonic seizure (1, 10.0)	Hypomania (5, 50.0), Jaw tingling (1, 10.0)	0
Hartmann et al. (2016)	6 (3)	NR	NR	NR
Holland et al. (2020)	9 (5)	NR	hypomania (1, 11.1)	NR
Huff et al. (2010)	10 (6)	0	Dysesthesia (1, 10.0), Transient agitation (4, 40.0), Suicidal thoughts (1, 10.0)	0
Huys et al. (2019)	20 (10)	Infection of IPG Pocket (1, 5.0); traction of IPG & cables (1, 5.0)	hypomanic state (1, 5.0); disinhibition (3, 15.0); lack of concentration (2, 10.0); transient loss of energy (1, 5.0); sleep disturbance (2, 10.0); significant weight gain (2, 10.0);	After cessation of stimulation: sudden increase in anxiety and anhedonia after acute cessation of stimulation (7, 35.0), suicidal thoughts (1, 5.0)
Islam et al. (2015)	8 (7)	NR	NR	Seizures (2, 25.0; one was infectious in origin & unrelated to surgery, the second related to poor diabetes control & hypoglycaemia)
Kahn et al. (2021)	5 (4)	0	Hypomania (3, 60.0); irritability (2, 40.0); jaw tightening (1, 20.0), pulling and tongue tingling (1, 20.0); insomnia (1, 20.0); sympathomimetic effects (1, 20.0); dysphoria (1, 20.0); aggression (1, 20.0); high pulse width (1, 20.0)	0
Li et al. (2020)	50 (24)	NR	NR	NR
Liebrand et al. (2019)	12 (2)	NR	NR	NR
Liebrand et al. (2021)	57 (16)	NR	NR	NR
Lopez-Sosa et al. (2021)	9 (5)	NR	NR	NR
Luyten et al. (2016)	24 (12)	misplacement of electrode/intraoperative correction (1, 4.2); rash iodine alcohol (1, 4.2); uncomfortable feeling around extension cables (12, 50.0), Pain around IPG (7, 29.2), Painful luxation IPG below ribs (3, 12.5), Skin infection (1, 4.2), Local transient inflammation of suture after IPG placement (1, 4.2)	Memory complaints (16, 66.7), Disinhibition (12, 50.0), Increased assertiveness (12, 50.0), Logorrhoea (10, 41.7), Hyperactivity (10, 41.7), Hypomania (4, 16.7), Confusion (4, 16.7), Patient smells something transiently (7, 29.2), Paraesthesia, pain or twitches in cheek/jaw & teeth grinding (6, 25.0), Transient perseveration in foreign language (1, 4.2), Micrographia (1, 4.2), Decreased libido (7, 29.2), increased libido (4, 16.7), Ejaculation problems (5, 20.8), Erection problems (4, 16.7), Diarrhoea (3, 12.5), Slow gastric emptying (1, 4.2), Fatigue (18, 75.0), Cough (2, 8.3)	Weight increase (13, 54.2), Weight decrease (10, 41.7), Insomnia (13, 54.2), Hypersomnia (7, 29.2), Vivid dreams (2, 8.3), Nightmares (1, 4.2), Family problems (13, 54.2), Irritability (16, 66.7), Tension/nervousness (13, 54.2), Apathy (7, 29.2), Aggressivity (6, 25.0), Derealisation/depersonalisation (4, 16.7), Transpiration (7, 29.2), nausea (7, 29.2), Tremor (6, 25.0), Disturbed Balance (5, 20.8), palpitations (5, 20.8), Increased ticks (3, 12.5), Transient urinary incontinence (3, 12.5), Nose bleed (2, 8.3), Body odour change (1, 4.2), song stuck in head (1, 4.2)
Mallet et al. (2008/2019)	16 (10)	Infection (2, 12.5), Intracerebral haemorrhage (1, 6.3), Clumsiness and diplopia with perielectrode oedema (1, 6.3)	Hypomanic state (3, 18.8), Anxiety (2, 12.5), Dyskinesia with impulsivity (1, 6.3), Facial asymmetry (1, 6.3), Dysarthria (1, 6.3), Dysphagia (1, 6.3), Walking disability (1, 6.3)	Urinary infection (1, 6.3), Nocturnal enuresis (1, 6.3) Diabetes Mellitus (1, 6.3)
Mosley et al. (2021)	9 (5)	DBS electrode migration (1, 11.1), infection of IPG (1, 11.1)	Persistent psychiatric problems (5 times non-responding participant, 2 in another participant’ 2, 22.2)	0
Naesstrom et al. (2021)	11 (4)	lack of hair regrowth at incision site (1, 9.1), Transient post-operative headache (1, 9.1)	Skin infection after accident with reimplantation (1, 9.1), anxiety (6, 54.5), insomnia (4, 36.4), fatigue (2, 18.2), hypomania (2, 18.2), nightmares (1, 9.1), weight gain (2, 18.2), medical intoxication/suicide attempt (14x in one patient; 1, 9.1)	0
Ooms et al. (2014)	16 (7)	NR	NR	NR
Parvaresh-Rizi et al. (2022)	4 (0)	IPG infection (1, 25.0) electrode migration (1, 25.0)	Suicidal thoughts (2, 50.0), Anxiety & depression symptoms (1, 25.0)	0
Raymaekers et al. (2017)	24 (12)	NR	One patient had repeated hypomanic episodes after battery replacements (1, 4.2) and also opted to undergo a capsulotomy procedure	NR
Sildatke et al. (2021)	15 (6)	NR	NR	NR
Tsai et al. (2012)	4 (4)	NR	Smell (3, 75.0), Chest vibration (1, 25.0), Chest tightness (2, 50.0), Dizziness (2, 50.0), Nausea (2, 50.0), Heat (2, 50.0), Increased sexual drive (2, 50.0)	NR
Tsai et al. (2014)	4 (4)	0	Hypomania episodes after several weeks of DBS stimulation (2, 50.0) Transient hypomania-like syndrome during DBS initial programming (1, 25.0) Allergic reaction to implantation of the pulse generator in the chest (1, 25.0) Vertigo (1, 25.0)	0
Tyagi et al. (2019)	6 (5)	0	Hypomania within hours after stimulation - amSTN (2, 33.3), VC/VS (2, 33.3), COMB phase (3, 50.0) - remedied with adjustment Sustained hypomania-like symptoms in optimum phase (1, 16.7)	VC/VS battery became depleted and was replaced once in 3 patients and twice in 1 patient (4, 66,7)
vanderVlis et al. (2021)	8 (2)	Minor infection due to surgery (2, 25.0), minor mechanical adverse effect (1, 12.5)	Transient complaints of hypomanic symptoms (4, 50.0) Severe wound infection from previous VC/VS implantation which had to be removed -> motor side effects (1, 12.5)	0
Voon et al. (2018)	12 (4)	NR	NR	NR
Welter et al. (2011)	12 (7)	NR	NR	NR
Widge et al. (2022)	8 (NR)	NR	Hypomania (3, 37.5)	NR
Winter et al. (2021)	6 (4)	Batteries discharged twice (1, 16.7)	NR	NR

In Table 3, a detailed summary of DBS-related complications reported by OCD patients in all included studies in this systematic review (*n* = 35) is shown. The following variables were extracted: sample size (of which males), surgery related complications (n), stimulation related complications (n) and long-term complications (n). Implantable Pulse Generator (IPG), No data reported (NR), Yale-Brown Obsessive Compulsive Score (Y-BCOS).

### Study characteristics

**Fig. 2:**
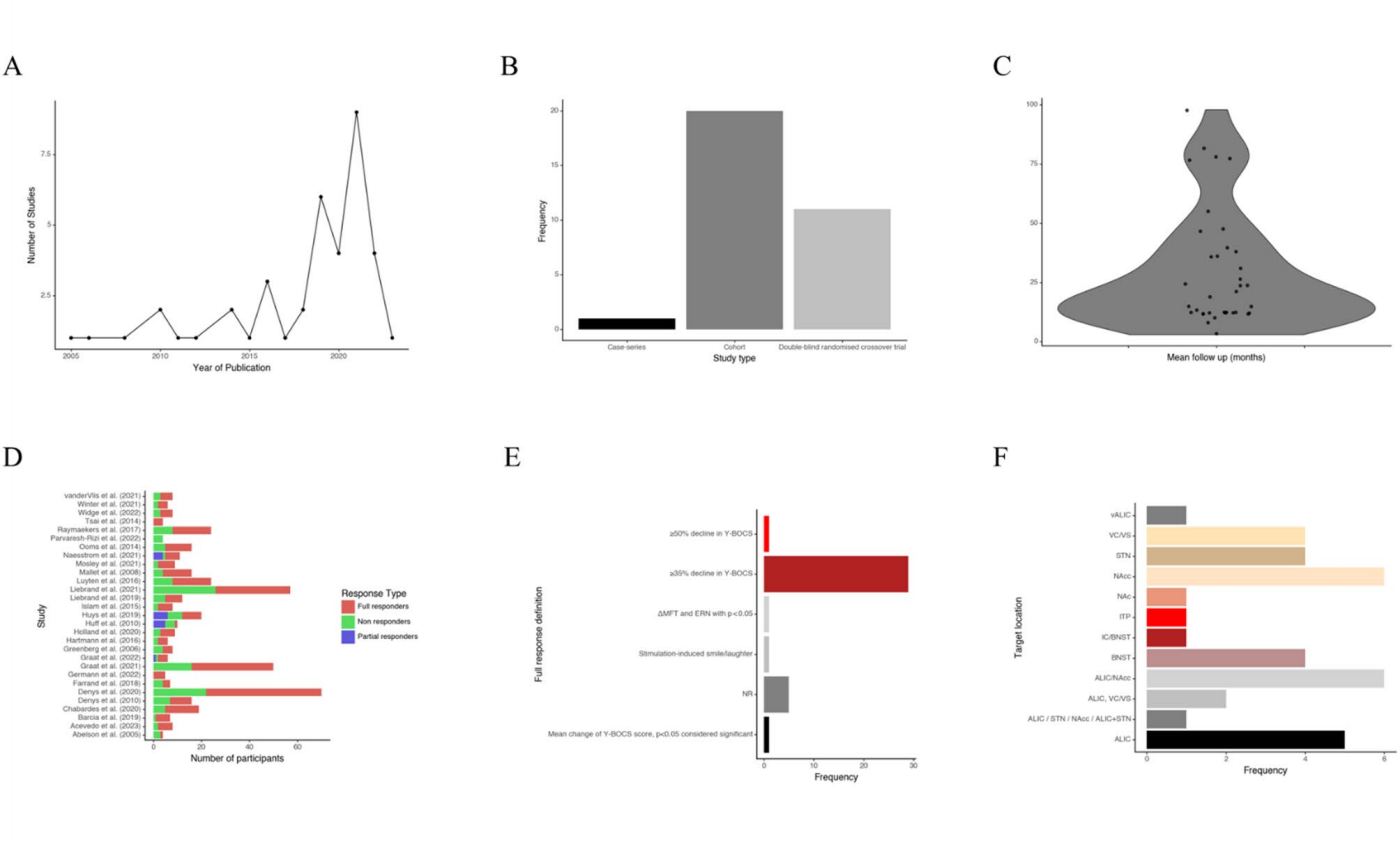
**A** depicts a line plot displaying the yearly trend of publications between 2005 and 2022. **B** showcases a bar chart that enumerates the frequency of the different types of studies incorporated in this review: case-series (*n* = 8), cohort studies (*n* = 24), and double-blind randomised controlled trials (*n* = 10). **C** offers a violin plot revealing the distribution of mean follow-up durations among the included studies. The width of the plot at various points indicates the relative number of studies with specific follow-up periods, highlighting the range, median, and distribution density of these durations. **D** illustrates a stacked bar chart representing the count of participants in each study, categorised as responders, partial responders, and non-responders. **E** showcases a bar plot that highlights the different definitions employed for determining a “full response” in the studies. MFT refers to conflict-related medial frontal theta, a brainwave pattern from the medial frontal cortex (MFC) detected by EEG that is associated with focussed attention and cognitive control. Error-related negativity (ERN) is an EEG pattern generated in the posterior MFC. Significant presence of these patterns (*p* < 0.05) was used by one study (Sildatke et al.) to define responders to DBS. The height of each bar signifies the frequency of studies adopting that specific definition for a full response. **F** visualises a bar graph representing the frequency of various target locations utilised for deep brain stimulation in the examined studies, with a clear demarcation of each target’s popularity.

All studies included in this systematic review (*n* = 41) are characterised in Fig. 2. Firstly, Fig. 2A demonstrates an increasing research interest from 2005 to 2022. Figure 2B shows that the majority of the research on the topic comprises cohort studies (*n* = 24), followed by randomised controlled trials (*n* = 10) and case series (*n* = 8). Figure 2C’s violin plot suggests that most studies have a mean follow-up duration clustered around 15 months post-DBS implantation. Figure 2D details the varied response outcomes of participants. Figure 2E depicts that the most common definition for a “full response” involved a ≥ 35% decline in the Yale-Brown Obsessive-Compulsive Scale (Y-BOCS) score. Lastly, Fig. 2F indicates multiple target locations for deep brain stimulation, with VC/VS being the most frequently chosen.

### Demographic patient factors

The examination of demographic factors in relation to treatment outcomes for OCD patients undergoing DBS yielded mixed findings. Most studies did not observe a pronounced variation based on gender. However, a unique observation by Chabardes et al.^17^ highlighted a trend where the majority of responders were females, while all non-responders were males. Huys et al.^23^, on the other hand, elucidated that there was no gender or age distinction influencing treatment outcomes. This perspective aligned with the findings by Naesstrom et al.^30^ and Raymaekers et al.^33^.

### Symptom predominance

Of the included literature, 18 studies^17,18,20,21,24,25,29,30,32,35–37,40,42,46–48,51^ examined the potential role of OCD symptom predominance in determining DBS response. In this context, symptom predominance refers to the primary symptom domains reported across study cohorts, reflecting the most pronounced obsessive-compulsive themes exhibited by patients rather than overall severity or comorbid conditions. The primary symptoms highlighted were contamination, checking, and intrusive thoughts. Barcia et al.^47^ demonstrated that mapping individual symptom-specific brain connectivity using fMRI and DTI can optimise DBS targeting, with the best stimulation site differing between patients based on their symptom-provoked activation networks. Denys et al.^18^, in contrast, identified symmetry, hoarding, and perfectionism as negative predictors of DBS response, potentially linked to poorer insight, as measured by the Brown Assessment of Beliefs Scale (BABS). In line with this, Graat et al. reported that preserved insight at baseline was associated with favourable clinical response following DBS, while insight alone did not demonstrate independent predictive value in univariate analyses^42,43^. Together, these findings suggest that insight may modulate the relationship between specific symptom dimensions and DBS outcomes, rather than acting as a standalone predictor. Moreover, these studies highlight that both neuroanatomical targeting and clinical phenotype play crucial roles in DBS response variability. A personalised DBS approach integrating neural circuit mapping with patient-specific symptomatology may enhance treatment precision and outcomes.

### Disease severity and duration

Among the studies reviewed, a subset (*n* = 3) probed the influence of preoperative OCD severity on subsequent treatment outcomes. Huys et al.^23^ Raymaekers et al.,^33^ and Baldermann et al.^16^ found no statistically significant correlation between pre-surgical Y-BOCS scores and treatment efficacy. Similarly, Baldermann et al.^16^ found no association between preoperative symptom intensity and postoperative treatment outcomes (*r*=−0.152, *p* = 0.499). Graat et al.^43^ found later onset of OCD to be associated with improved symptoms (*p* = 0.023) as well as good insight into illness as determined by the BABS scale. Ooms et al.^31^ also investigated the correlation between disease duration and effectiveness of DBS: their analysis revealed that longer disease duration was inversely related to post-DBS enhancements in the social dimension of the WHO-QOL-BREF (*r* = − 0.587, *p* = 0.035).

### Co-morbidities

Amongst a predominant portion of the included studies (*n* = 32), depression metrics emerged as a recurrent theme. Raymaekers et al.^33^ found that heightened Beck’s Depression Inventory (BDI) scores, indicative of severe co-morbid depression, lowered the probability of a favourable response (*p* = 0.049). On the contrary, the data of Holland et al.^22^ shows that co-morbid major depressive disorder (MDD) could enhance response rates.

### Treatment efficacy and safety

Treatment efficacy of deep brain stimulation (DBS) varied between studies. Several studies demonstrated marked improvement post-intervention, with response rates ranging from 25%^50^ to 100%^36^. The specific adverse events from individual studies can be found in Table 3. Stimulation side-effects were prevalent across the cohort and were reported in 26 studies. The most common side-effect from stimulation was transient hypomania. Out of the studies analysed, 22 studies provided data on surgical complications, of which 15 reported surgical complications. Surgical site infections and hardware-related complications were the most frequent, being reported in 9 studies^17,23,41,43,49,51,52,54,56^. However, the most severe adverse events, reported by six studies^17,23,30,32,49,54^ included suicidal ideation, attempts and successful suicide. Where reported, these events occurred in patients with severe, treatment-refractory OCD and significant psychiatric comorbidity. Importantly, the included studies did not consistently attribute these events directly to DBS, and in most cases causal relationships could not be determined from the available data.

### Meta-analysis

#### Exploratory, correlational and effect size analysis

**Fig. 3:**
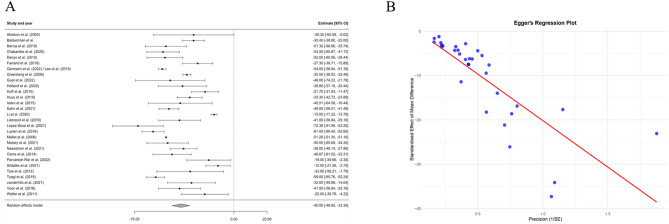
Forest plots displaying the effects of interventions on Y-BOCS (Yale-Brown Obsessive-Compulsive Scale) scores. Figure 3A presents the percentage change in Y-BOCS scores across various studies, with the estimate and 95% confidence intervals (CIs) indicating the mean difference from baseline. The panel includes a summary estimate at the bottom, which represents the pooled effect size calculated using a random effects model. The dashed vertical line denotes the line of no effect, emphasising the direction and magnitude of the intervention effects across studies. The main finding, summarised by the random effects model, suggests a consistent treatment effect with no apparent outlier studies. Figure 3B displays Egger’s Regression Plot for Publication Bias, assessing the funnel plot’s symmetry to investigate potential publication bias. The plot shows the standardised mean difference plotted against the inverse of the standard error, which helps to visualise the relationship between study size and treatment effect. A linear regression line (in red) suggests the extent to which effect sizes are influenced by study precision, aiding in the identification of asymmetry in the meta-analysis, which can be indicative of publication bias. The Egger’s regression test indicates no significant publication bias (estimate = −20.9631, SE = 0.7956, *p* = 0.6810).

**Fig. 4:**
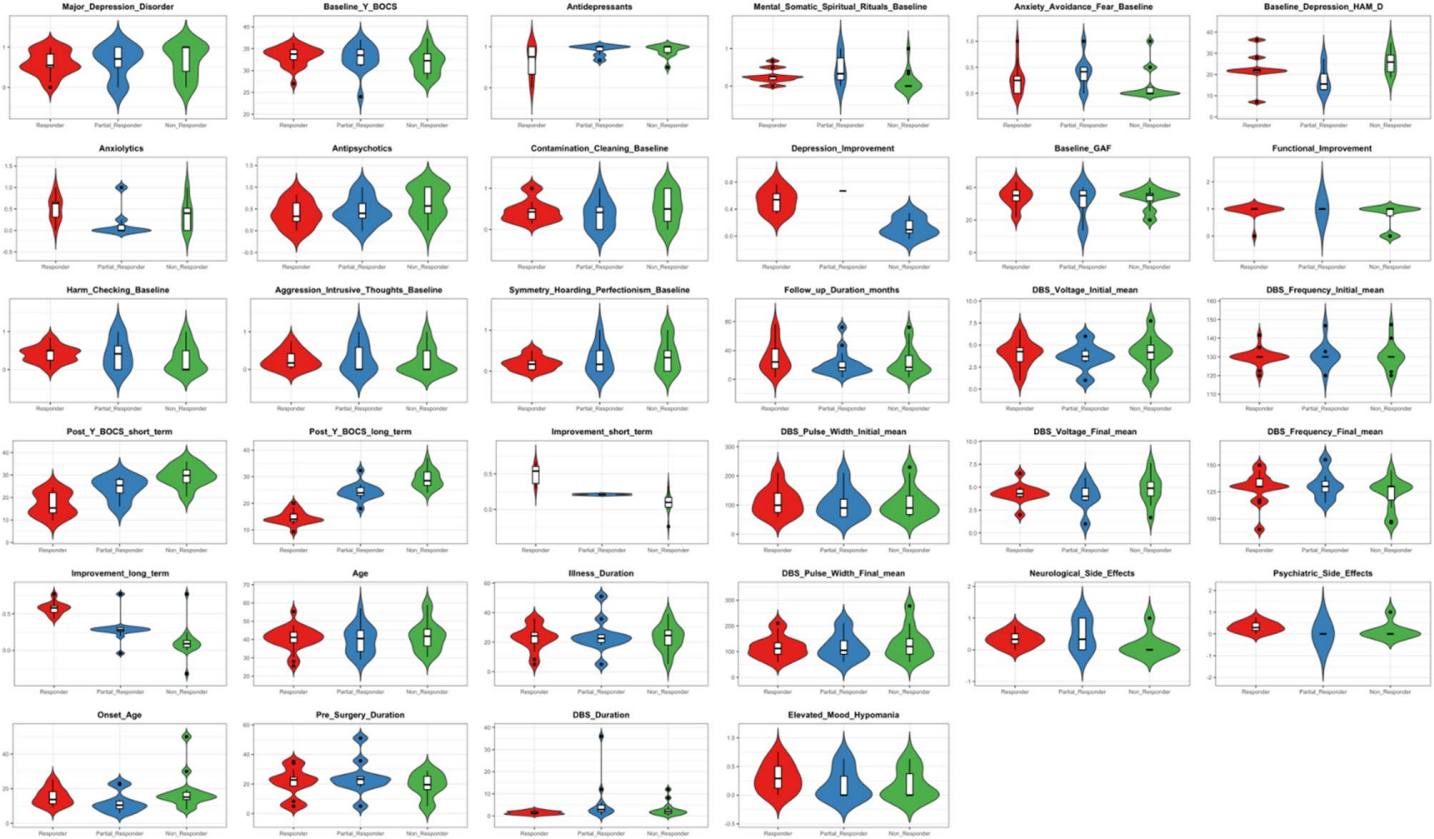
A grid of violin plots comparing patient and disease characteristics between three groups: responders, partial responders, and non-responders to DBS treatment in OCD. Each violin plot captures the distribution of a specific variable, with its width denoting the frequency of the data. Overlaid on each plot is a box plot, signifying the median and interquartile range for each characteristic. The colour of the violins differentiates the three groups, and the title above each highlights the variable under scrutiny. This comprehensive visualisation allows for an in-depth analysis of potential factors influencing DBS treatment outcomes in OCD. Overall, the key differentiating factors between responders and non-responders were the use of antidepressants and specific OCD symptomatology including symmetry, hoarding, and perfectionism, whereas high baseline Y-BOCS scores and elevated mood/hypomania were the differentiating factors between responders and partial responders.

**Fig. 5:**
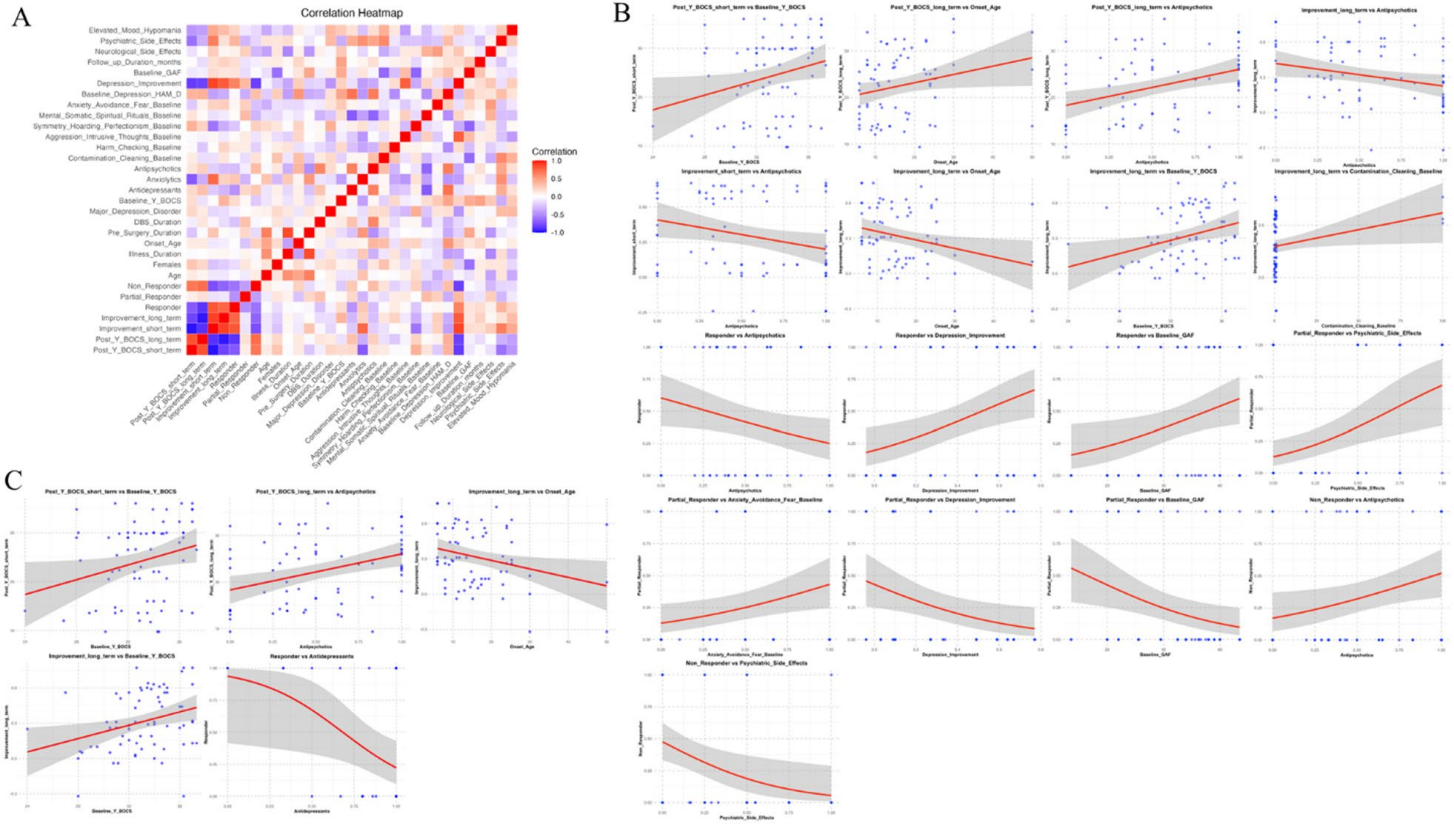
Encompasses a tripartite analysis visualising correlations and predictive relationships relevant to DBS treatment outcomes, employing both unimputed and PMM-imputed datasets. Figure 5A displays a correlation heatmap elucidating the correlation coefficients between all pairs of numeric variables contained within the unimputed dataset. T Each square in the matrix corresponds to a correlation coefficient derived from a pairwise comparison between two variables, the identities of which are defined by the corresponding row and column labels. The colour gradient of each square is reflective of the magnitude and direction of the correlation - from a strong negative (purple) correlation through no correlation (white) to a strong positive (red) correlation. Despite these visual correlations, none reached a level of statistical significance (all p-values > 0.05), as determined by the conventional threshold for statistical significance. Figure 5B shows a set of scatter plots delineating the relationships between outcome variables and corresponding statistically significant predictors, as identified by multivariate linear regression models and multivariate logistic regressions. These models were fit on an unimputed dataset. Each scatter plot has been constructed with the predictor variable represented along the x-axis and the dependent outcome variable along the y-axis. Individual data points are depicted as blue markers, and a best-fit regression line, demonstrating the trend and magnitude of the relationship, is drawn in bold red. The shaded region around the regression line corresponds to standard error, thus providing an indication of the variability or uncertainty inherent in the predicted relationship. Figure 5C shows a set of scatter plots delineating the relationships between outcome variables and corresponding statistically significant predictors, as identified by multivariate linear regression models and multivariate logistic regressions. These models were fit on a dataset subjected to predictive mean matching (PMM) to address missing data. Each scatter plot is arranged with the predictor variable on the x-axis and the respective binary outcome variable on the y-axis. The individual data points, extracted from the mean-imputed data, are represented as blue markers. Their distribution reveals the fundamental patterns between variables. A fitted logistic regression line, depicted in bold red, portrays the probability of the outcome as a function of the predictor variable. Unlike in linear regression, this line does not depict a linear relationship but a logistic one, suitable for binary outcomes. The shaded area surrounding the regression line demonstrates the standard error, serving as an estimate of the precision of the predicted relationship. It represents the degree of variability or uncertainty associated with the predicted outcome. Overall, the use antidepressants and symmetry/hoarding/perfectionism symptomology were found to be negative predictors, and high Y-BOCS baseline scores, elevated mood/hypomania were positive predictors of DBS response in OCD patients.

The forest plot in Fig. 3A^3,8,16–20,22–25,27,29–32,34,35,37,38,45–47,49–54^ highlights the percentage change in Y-BOCS scores post-DBS (−40.00, Y-BOCS % Change; 95% CI: −46.65 - −35.34), without the presence of outliers. Similarly, Supplementary Fig. 3 provides a forest plot of the absolute change in Y-BOCS scores, which also supports the finding of a positive intervention effect (−17.86 Y-BOCS Absolute Change; 95% CI: −21.24 - −14.48). As shown in Supplementary Fig. 4, a positive effect favouring treatment was found across all study designs including case-series (−36.29 Y-BOCS % Change; 95% CI: −49.87 to −22.72), cohort studies (−41.26 Y-BOCS % Change; 95% CI: −48.73 - −33.79) and RCTs (−42.97 Y-BOCS % Change; 95% CI: −51.94 - −34.01). This revealed a consistent effect in favour of treatment regardless of study design (Supplementary Fig. 4). Expectedly, Egger’s Regression Plot for publication bias (Fig. 3B) reveals no significant publication bias (*p* > 0.05). An exploratory visual descriptive analysis distinguished patterns of patient and disease characteristics among responders, partial responders, and non-responders (Fig. 4). Baseline features such as symmetry, hoarding, perfectionism, and contamination-cleaning, as well as major depression disorder and use of antipsychotics were more prominent in non-responders. In Fig. 5A, the correlation heatmap reveals distinct patterns among numeric variables. Baseline assessments, notably elevated mood and anxiolytics use correlate with DBS outcomes. However, none of the correlations reached statistical significance (*p* > 0.05).

#### Chi-squared, ANOVA and Tukey’s HSD analysis

No difference was found between response groups (Responder, Partial Responder, and Non-Responder) in terms of target location (Supplementary Table 6) (*p* > 0.05) using Chi-squared analysis. One-way ANOVA analyses and Tukey’s HSD were performed, the results of which are reported in Supplementary Tables 7 and 8. Significant differences between the three response groups were found only for the use of antidepressants (*p* < 0.05).

#### Univariate and stepwise multivariate regression analyses

Univariate linear regression analyses of the unimputed dataset (Supplementary Table 9) found that anxiolytic use was significantly associated with both lower short-term Y-BOCS scores and greater short-term improvement, suggesting a reduction in symptom burden (*p* < 0.001 for both models). Baseline traits of symmetry, hoarding, and perfectionism were negatively associated with short-term improvement (*p* = 0.0316), while higher baseline Y-BOCS scores significantly predicted long-term improvement (*p* = 0.0051). These associations were preserved in the stepwise multivariate model.

Analyses of the imputed dataset (Supplementary Table 11) revealed that baseline Y-BOCS scores were again positively associated with both short-term and long-term improvements (*p* = 0.0341 and *p* = 0.0189, respectively), and antipsychotic use was negatively associated with both short-term and long-term improvement (*p* = 0.0351 and *p* = 0.0264, respectively). Stepwise multivariate regression again retained these predictors. Additionally, contamination-cleaning symptoms at baseline were weakly associated with poorer long-term improvement (*p* = 0.0542).

Logistic regression analysis of categorical response outcomes (Supplementary Table 12) showed that antipsychotic use was negatively associated with responder status (*p* = 0.0394) and positively associated with non-response (*p* = 0.0307). Baseline anxiety/avoidance/fear symptoms were significantly associated with partial responder status (*p* = 0.0207). These results were retained in stepwise multivariate models.

Univariate linear regression analyses of the unimputed dataset showed significant (*p* < 0.05) negative associations between the use of anxiolytics and short-term Y-BOCS scores (absolute and percentage change), indicating that the use of anxiolytics is associated with a decrease in symptom severity in the short term (Fig. 5B, Supplementary Table 9). For short-term Y-BOCS percentage improvement, anxiolytics were positively associated, while baseline traits such as symmetry, hoarding, and perfectionism were negatively associated. In terms of long-term Y-BOCS percentage improvement, a higher baseline Y-BOCS score was positively associated with long-term Y-BOCS percentage improvement. Univariate logistic regression (Responder versus Partial Responder versus Non-Responder) of the unimputed dataset identified a negative association between antidepressant use and DBS response (Supplementary Table 10). These analyses using the imputed dataset are available in Fig. 5C, and Supplementary Tables 11 and 12.

#### Final co-variate boosted multivariate regression analyses

**Fig. 6:**
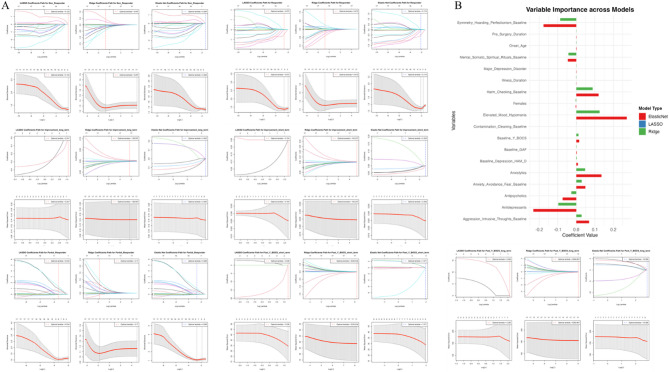
**A** presents the path plots and cross-validation plots from three different regularised regression techniques: LASSO (Least Absolute Shrinkage and Selection Operator), Ridge, and Elastic Net, applied to the predictors of response to deep brain stimulation (DBS) for obsessive-compulsive disorder (OCD). Each row of plots corresponds to a different imputed dataset used in the analysis. The left column shows the LASSO coefficients path plot for each dataset, with the logarithm of lambda on the x-axis and the coefficients of the predictors on the y-axis. The rainbow-colored lines represent the trajectory of each predictor’s coefficient as the regularisation penalty increases; coefficients shrink toward zero, with some reaching exactly zero, indicating that LASSO has eliminated them from the model. The dotted vertical line marks the lambda value chosen by cross-validation as the optimal trade-off between complexity and accuracy. The middle column contains the Ridge coefficients path plot. Unlike LASSO, Ridge regression does not set coefficients to zero but shrinks them toward zero as the penalty increases. The path of each coefficient is represented as lambda varies, with no coefficients reaching zero. The right column displays the Elastic Net coefficients path plot, which combines features from both LASSO and Ridge. It shrinks coefficients like Ridge but can also set some coefficients to zero like LASSO, as indicated by the lines dropping to the horizontal axis. Below each coefficients path plot is a cross-validation plot for the corresponding regularisation method, featuring the mean squared error (MSE) on the y-axis and the logarithm of lambda on the x-axis. The red line represents the average MSE across all folds of cross-validation, and the grey bands indicate the upper and lower bounds of the standard error. The dotted vertical lines again signify the optimal lambda value that minimises the MSE. **B** shows the Variable Importance across Models plot. This bar chart provides a comparison of the magnitude and direction of standardised coefficients for the most important predictors as determined by the LASSO, Ridge, and Elastic Net models. The variables are listed on the y-axis, and the standardised coefficient values are on the x-axis, extending to the left for negative associations. Each predictor’s impact is color-coded according to the model—red for LASSO, blue for Ridge, and green for Elastic Net. Negative coefficient values suggest that an increase in the predictor is associated with a decrease in the likelihood of a positive DBS response. To summarise, the most important variables were found to be: symmetry/hoarding/perfectionism baseline, elevated mood/hypomania baseline symptomology, baseline Y-BOCS scores, Anxiety/Avoidance/Fear baseline symptomology, antipsychotic use, antidepressant use and aggressive/intrusive thoughts baseline symptomology.

Three different regularised regression techniques were used to find the most important co-variates to predict our OCD outcome variables: LASSO, Ridge, and Elastic Net (Fig. 6A-B). A final multivariate linear regression analysis was computed using the final co-variates (symmetry/hoarding/perfectionism baseline, elevated mood/hypomania baseline symptomology, baseline Y-BOCS scores, Anxiety/Avoidance/Fear baseline symptomology, antipsychotic use, antidepressant use and aggressive/intrusive thoughts baseline symptomology) (Table 4). In the final multivariate model, antipsychotic use was a significant negative predictor of long-term Y-BOCS reduction (*p* = 0.0138), whereas higher baseline Y-BOCS scores significantly predicted improvement in the long term (*p* = 0.0075). The findings of the final multivariate linear regression analysis were confirmed by the final multivariate logistic regression analysis (Table 5).

**Table 4:** Final mixed-effects multivariate linear regression model of continuous outcome variables of DBS response (PMM).

Explanatory variable	Outcome variable	Estimate	SE	t-statistic	*p*-value
Baseline_Y_BOCS	Post_Y_BOCS_short_term	0.793724	0.397923	1.994667	0.050553
Antidepressants	Post_Y_BOCS_short_term	−2.12754	2.767801	−0.76867	0.445053
Antipsychotics	Post_Y_BOCS_short_term	3.229349	2.897656	1.114469	0.26945
Aggression_Intrusive_Thoughts_Baseline	Post_Y_BOCS_short_term	−0.65167	2.798219	−0.23289	0.81663
Symmetry_Hoarding_Perfectionism_Baseline	Post_Y_BOCS_short_term	−0.1149	2.641043	−0.04351	0.96544
Anxiety_Avoidance_Fear_Baseline	Post_Y_BOCS_short_term	−0.81376	2.759422	−0.2949	0.769069
Elevated_Mood_Hypomania	Post_Y_BOCS_short_term	−5.4773	4.050991	−1.35209	0.181338
Baseline_Y_BOCS	Post_Y_BOCS_long_term	−0.29312	0.339441	−0.86354	0.391222
Antidepressants	Post_Y_BOCS_long_term	1.347638	2.361018	0.570787	0.570241
Antipsychotics	Post_Y_BOCS_long_term	6.267762	2.471789	2.535719	0.0138 *
Aggression_Intrusive_Thoughts_Baseline	Post_Y_BOCS_long_term	−0.21204	2.386966	−0.08883	0.929505
Symmetry_Hoarding_Perfectionism_Baseline	Post_Y_BOCS_long_term	2.136308	2.25289	0.948252	0.346743
Anxiety_Avoidance_Fear_Baseline	Post_Y_BOCS_long_term	2.353719	2.353871	0.999936	0.321292
Elevated_Mood_Hypomania	Post_Y_BOCS_long_term	−5.32749	3.455619	−1.54169	0.128321
Baseline_Y_BOCS	Improvement_short_term	0.018457	0.014423	1.279654	0.205513
Antidepressants	Improvement_short_term	−0.03203	0.100323	−0.31924	0.750635
Antipsychotics	Improvement_short_term	−0.16492	0.105029	−1.57023	0.121534
Aggression_Intrusive_Thoughts_Baseline	Improvement_short_term	0.02058	0.101425	0.202906	0.839884
Symmetry_Hoarding_Perfectionism_Baseline	Improvement_short_term	−0.0087	0.095728	−0.09089	0.927879
Anxiety_Avoidance_Fear_Baseline	Improvement_short_term	−0.10259	0.100019	−1.02575	0.309059
Elevated_Mood_Hypomania	Improvement_short_term	0.240401	0.146834	1.637232	0.106731
Baseline_Y_BOCS	Improvement_long_term	0.033298	0.01204	2.765707	0.007505 **
Antidepressants	Improvement_long_term	−0.05673	0.083743	−0.67738	0.500726
Antipsychotics	Improvement_long_term	−0.12312	0.087671	−1.40432	0.165296
Aggression_Intrusive_Thoughts_Baseline	Improvement_long_term	0.004462	0.084663	0.052698	0.958145
Symmetry_Hoarding_Perfectionism_Baseline	Improvement_long_term	−0.06391	0.079907	−0.79984	0.426909
Anxiety_Avoidance_Fear_Baseline	Improvement_long_term	−0.09442	0.083489	−1.13091	0.262521
Elevated_Mood_Hypomania	Improvement_long_term	0.155085	0.122567	1.265309	0.210573

In Table 4, the results of the final mixed-effects multivariate linear regression model of continuous outcome variables of DBS response using the predictive mean matching (PMM) imputed dataset are shown. Single asterisks (*) indicate p-values less than 0.05, while double asterisks (**) denote p-values less than 0.01, signifying statistical significance. The table delineates the regression coefficient (Estimate), standard error (SE), t-statistic, and p-value for each relationship studied.

**Table 5:** Final mixed-effects multivariate logistic regression model of categorical outcome variables of DBS response (PMM).

Explanatory variable	Outcome variable	Estimate	SE	z-statistic	*p*-value
Baseline_Y_BOCS	Responder	0.257165	0.129803	1.981195	0.047569 *
Antidepressants	Responder	−1.05071	0.785963	−1.33684	0.181275
Antipsychotics	Responder	−1.2919	0.804733	−1.60538	0.108411
Aggression_Intrusive_Thoughts_Baseline	Responder	1.064965	0.795251	1.339155	0.18052
Symmetry_Hoarding_Perfectionism_Baseline	Responder	−0.21191	0.729695	−0.29041	0.771499
Anxiety_Avoidance_Fear_Baseline	Responder	−0.70817	0.76832	−0.92172	0.356677
Elevated_Mood_Hypomania	Responder	0.108403	1.106873	0.097936	0.921983
Baseline_Y_BOCS	Partial_Responder	−0.04164	0.119638	−0.34803	0.727816
Antidepressants	Partial_Responder	−0.03667	0.848893	−0.04319	0.965546
Antipsychotics	Partial_Responder	−0.52057	0.914536	−0.56921	0.569211
Aggression_Intrusive_Thoughts_Baseline	Partial_Responder	−0.3379	0.896103	−0.37707	0.706119
Symmetry_Hoarding_Perfectionism_Baseline	Partial_Responder	0.243708	0.823827	0.295824	0.767364
Anxiety_Avoidance_Fear_Baseline	Partial_Responder	1.497376	0.834046	1.795316	0.072603
Elevated_Mood_Hypomania	Partial_Responder	1.344653	1.170602	1.148685	0.250686
Baseline_Y_BOCS	Non_Responder	−0.20433	0.120623	−1.69392	0.09028
Antidepressants	Non_Responder	1.464692	0.921747	1.589039	0.112052
Antipsychotics	Non_Responder	1.751215	0.886448	1.97554	0.048207 *
Aggression_Intrusive_Thoughts_Baseline	Non_Responder	−0.85228	0.835704	−1.01983	0.307808
Symmetry_Hoarding_Perfectionism_Baseline	Non_Responder	−0.05897	0.756857	−0.07792	0.937891
Anxiety_Avoidance_Fear_Baseline	Non_Responder	−0.67802	0.822263	−0.82458	0.409612
Elevated_Mood_Hypomania	Non_Responder	−1.5868	1.255044	−1.26434	0.206109

In Table 5, the results of the final mixed-effects multivariate logistic regression model of continuous outcome variables of DBS response using the predictive mean matching (PMM) imputed dataset are shown. Single asterisks (*) indicate p-values less than 0.05, while double asterisks (**) denote p-values less than 0.01, signifying statistical significance. The table delineates the regression coefficient (Estimate), standard error (SE), z-statistic, and p-value for each relationship studied.

#### Sensitivity analysis

In a sensitivity analysis adjusting for stimulation target, aggression and intrusive thoughts symptomology emerged as a significant positive predictor of DBS response (*p* = 0.0475) in both mixed-effects linear and logistic regression models (Supplementary Tables 13–14). Target location itself remained non-significant across all models (*p* > 0.05).

In a sensitivity analysis excluding studies with high or critical risk of bias higher baseline Y-BCOS scores significantly predicted DBS response across both linear and logistical models (*p* < 0.05). Additionally, the OCD symptomology phenotype of anxiety, avoidance and fear was associated with improved DBS response in the multivariate logistical regression mode result (*p* = 0.024) (Supplementary Tables 15 and 16).

## Discussion

Patients with treatment-resistant OCD are typically refractory to pharmacological interventions and prolonged cognitive behavioural therapy. In this subgroup of patients, DBS shows significant efficacy, with approximately two-thirds of patients showing > 50% improvement in Y-BOCS scores^47^. Indeed, similar results have previously led to calls for wider access to DBS treatment in OCD^4^. However, the efficacy of DBS remains variable, with efforts being made to identify the drivers of variability^8^. This systematic review and meta-analysis, which included 41 studies with a total of 589 patients, identifies key clinical predictors of response to DBS in treatment-resistant OCD, with the potential to aid future patient selection^57,58^..

Our meta-analysis of 296 OCD patients reveals that those with a higher initial Y-BOCS score tend to have better long-term outcomes with DBS. The presence of aggressive or intrusive thoughts, as well as anxiety, fear and avoidance symptomology at baseline, was also associated with favourable outcomes, particularly when adjusting for stimulation target. In contrast, the use of antipsychotics and antidepressants, as well as baseline symptom profiles marked by symmetry, hoarding, and perfectionism, were associated with poorer DBS response. These findings support the notion that distinct OCD clinical phenotypes, defined by symptom dimension and pharmacological context, may respond differentially to neuromodulatory intervention^59,60^..

Previous systematic reviews and meta-analyses have yielded mixed findings on clinical predictors of OCD DBS outcomes, but some clear patterns are emerging^61^. In an early landmark study, Denys et al. observed that patients with predominant symmetry/ordering or hoarding symptoms had significantly poorer DBS responses, a trend attributed to these subtypes’ often poorer insight^48^. Conversely, a 2015 meta-analysis by Alonso and colleagues found that later age at OCD onset and the presence of aggressive “intrusive” obsessions (e.g., sexual or religious themes) were associated with superior DBS outcomes^62^. Notably, most earlier studies did not identify baseline illness severity or medication status as significant outcome modifiers^23,62^. The current meta-analysis both supports and extends this literature: our finding that higher baseline Y-BOCS scores and aggressive/intrusive obsessional content predict greater post-DBS improvement is in line with the positive impact of sexual/religious obsession dimensions reported previously, while the identification of symmetry/hoarding/perfectionism symptoms as markers of poorer response echoes Denys et al.’s results^48^. Furthermore, the novel observation that concurrent antidepressant or antipsychotic use portends a weaker DBS response was not highlighted in prior reviews, suggesting that patients requiring these medications may represent a more refractory subset for whom DBS is less efficacious. This comparison with past evidence underscores that our findings largely corroborate earlier hints that OCD symptom dimensions matter, while introducing baseline severity and concurrent medication use as additional predictors of DBS treatment outcome in OCD.

Multiple neural networks have been implicated in the pathophysiology of OCD^54^. The cortico-striatal-thalamo-cortical circuit, particularly its integration of the orbitofrontal cortex, anterior cingulate cortex, and striatal structures, plays a critical role in the regulation of intrusive thoughts and maladaptive cognitive control. The association between baseline aggressive or intrusive thoughts and favourable DBS response observed in our study makes it plausible that pre-existing neurobiological features—such as heightened cortico-striatal connectivity, altered serotonergic and dopaminergic signalling, and distinct limbic-prefrontal network activation—may facilitate a more favourable neuromodulatory response. These factors could enhance cognitive flexibility and reduce pathological threat monitoring, thereby reinforcing the therapeutic effects of DBS. On the other hand, the sensorimotor circuit, linked with excessive habit formation, might be more related to the symmetry, hoarding, and perfectionism traits in OCD^55^. The fact that higher baseline scores for these traits adversely impact short and long-term improvements suggests that disruptions in this circuitry could reduce the efficacy of DBS treatments. The ventral and dorsal cognitive circuits, as well as the ventral affective circuit, also represent intriguing targets, given their roles in impaired response inhibition, executive dysfunction, and altered reward mechanisms respectively^55^. The overlap between these circuits and various OCD subtypes, coupled with our meta-analysis findings, underscores the notion that the neural basis of the disorder could influence therapeutic outcomes. Here, we provide preliminary evidence that certain preoperative phenotypes may be more suited to DBS therapy, which warrants further exploration^26,63,64^..

The differential DBS outcomes observed with concurrent antidepressant, antipsychotic, or anxiolytic use likely reflect how each drug modulates the cortico-striato-thalamo-cortical (CSTC) circuit and neurotransmitter milieu underlying OCD. Antidepressants—particularly serotonin reuptake inhibitors (SRIs)—enhance serotonin tone and rectify the serotonergic deficits thought to drive OCD, which in turn can normalise aberrant striatal dopamine signaling^65^. Notably, neuroimaging studies indicate that chronic SSRI therapy and DBS produce convergent effects on OCD circuitry, such as reducing hyperactivity and pathologic connectivity in orbitofrontal–striatal loops^66^. This serotonergic augmentation may thus synergise with DBS by priming frontostriatal networks for plasticity and facilitating processes like fear-extinction learning—an effect also promoted by DBS^65^. By contrast, antipsychotics (dopamine D2 receptor blockers) blunt dopaminergic transmission and are often used in refractory OCD to target a putative dopamine hyperactivity component. However, this dopaminergic dampening could counteract one mechanism of DBS—namely, the restoration of striatal dopamine balance and reward-circuit function, thereby potentially diminishing DBS-driven improvements in motivation or mood^67^. Finally, anxiolytic sedatives (e.g., benzodiazepines), through potentiation of GABA_A receptors, broadly inhibit neural excitability and acutely reduce anxiety. Such GABAergic enhancement might reinforce the local inhibitory effects of DBS (as high-frequency stimulation can recruit GABA interneurons to suppress pathological firing), yet excessive neuronal suppression and sedation might limit the patient’s engagement in adaptive cognitive-behavioural changes and interfere with the long-term fear-extinction and habit-reversal benefits that DBS confers^65^..

In summary, concurrent medications modulate key neurotransmitter systems—serotonin, dopamine, and GABA—that are intimately linked to OCD pathophysiology, thereby altering the neurophysiological substrate on which DBS acts. Consistent with this, evidence from clinical neuroimaging and neurochemical studies shows that SSRIs and DBS can both attenuate hyperactive fronto-striatal circuits, whereas dopamine-blocking antipsychotics may necessitate stronger DBS-driven network activation to overcome reduced striatal reactivity, and GABAergic anxiolytics provide symptomatic relief but may blunt the cognitive–affective remodelling that optimises DBS response. This mechanistic framework aligns with prior findings and helps explain why the efficacy of OCD-DBS may vary with the use of concomitant antidepressants, antipsychotics, or anxiolytics^65–67^.

A major limitation of this study is the inherent methodological variability in the current literature on DBS for OCD, with a large proportion of non-RCT evidence. Consequently, multiple studies were flagged for ‘moderate’ to ‘critical’ risk of bias. Another source of heterogeneity arises from a lack of consensus on anatomical targeting and stimulation parameters, which were not investigated in the present study. A significant limitation of our study is the absence of homogeneous data pools and clear outcome reporting guidelines in the existing literature on DBS for OCD. This inconsistency necessitated data imputation for our meta-regression analysis, a process undertaken to address gaps and disparities in the reported findings. This approach introduces potential biases, as imputed values are derived from statistical estimations rather than direct empirical evidence. This factor, alongside the observed variability in study designs and methodologies, underscores the challenges in synthesising a cohesive understanding of DBS efficacy in OCD treatment. The study’s reliance on a diverse array of clinical narratives, rather than uniform, specialised assessment tools, further contributes to the overall methodological variability, embedding a degree of subjectivity in our analysis. Different scales and scoring methods were used to characterise OCD sub-symptomatology, such as anxiety, fear and avoidance. However, as these measures were primarily analysed in a categorical framework rather than as continuous variables, we consider them broadly comparable for the purpose of this study, though further standardisation in future research would enhance comparability. The restricted availability of patient-level data also limited our ability to include neuroimaging-based explanatory variables, now recognised for their predictive value in treatment response^8^. Furthermore, the risks associated with suicide and death by suicide among DBS-treated OCD patients necessitate careful consideration^20,52,58,60^. Overall, reports of suicidality were heterogeneous and inconsistently contextualised across studies, often without detailed temporal or causal assessment, precluding definitive conclusions regarding any direct relationship between DBS and suicidal behaviour. Such events likely reflect the profound psychiatric burden and comorbidity characteristic of this population rather than a specific device-related effect.

## Conclusion

This meta-analysis of 296 patients found that higher baseline Y-BOCS scores significantly predicted improvement in the long term (*p* < 0.01), while antipsychotic use was a significant negative predictor of long-term response (*p* < 0.05). In univariate analyses, antidepressant use was negatively associated with DBS response (*p* < 0.05), and anxiolytic use was positively associated with short-term improvement (*p* < 0.05). Symmetry, hoarding, and perfectionism symptomology at baseline predicted reduced short-term improvement (*p* < 0.05), while aggression and intrusive thoughts emerged as a positive predictor of response when adjusting for stimulation target in the sensitivity model (*p* < 0.05). Additionally, in the multivariate analysis excluding studies with high risk of bias, anxiety, avoidance, and fear symptomology was also associated with improved DBS response (*p* < 0.05). Our findings suggest that there may be distinct phenotypes of OCD which respond differently to DBS. Further research with homogenous methodology and outcome reporting, as well as randomised control trials, are required to further elucidate these phenotypes and allow for more personalised and thus likely more effective DBS treatment strategies for all OCD patients.

Table 2 provides a comprehensive summary of both patient and disease characteristics for DBS-treated OCD patients as reported in all the included studies in this systematic review (*n* = 35). The table elucidates information on the predominant symptom dimension, the mean pre-intervention Y-BOCS score, the mean post-intervention Y-BOCS score, the mean percentage improvement in the Y-BOCS score, and the number of responders along with the percentage given in brackets. Abbreviations used within the table are defined as follows: Anterior Limb of Internal Capsule (ALIC), Cognitive Behavioural Therapy (CBT), No data Reported (NR), Subthalamic Nucleus (STN), and Yale-Brown Obsessive-Compulsive Score (Y-BOCS).

## Supplementary Information

Below is the link to the electronic supplementary material.

Supplementary Material 1

## Data Availability

All relevant data supporting the findings of this study can be accessed within the Supplementary Digital Content attached to the article. Additionally, the R code and Python code, as well as a comprehensive dataset used for the meta-analysis is freely available and can be retrieved from two public GitHub repositories. To ensure transparency and replicability of the research, the repositories includes both raw data and processed data utilized in the study. Please visit the following link for the R code and dataset access: https://github.com/santhoshgthava/OCDPredictorsMA https://github.com/santhoshgthava/OCDPredictorsMA. Please visit the following link for the Python code and dataset access: https://github.com/dscr00/ocd\_dbs. We strongly encourage researchers and interested parties to utilize these resources in their own investigations and analyses.

## Abbreviations

ALIC: Anterior Limb of Internal Capsule
ATR: Anterior Thalamic Radiation
amSTN: Anteromedial subthalamic nucleus
ANOVA: Analysis of variance
BDI: Beck’s Depression Inventory
BNST: Bed Nucleus of Stria Terminalis
CBT: Cognitive behavioural therapy
CT: Computed tomography
DBS: Deep Brain Stimulation
DWI: Diffusion-weighted imaging
EEG: Electroencephalogram
ERN: Error-related negativity
ERP: Exposure and response prevention
fMRI: Functional magnetic resonance imaging
FR: Full response
IPG: Implantable pulse generator
MCAR: Missing-completely-at-random
MDD: Major depressive disorder
MFT: Conflict-related medial frontal theta
NAcc: Nucleus Accumbens
NICE: National institute for Health and Care Excellence
NR: No data reported
OCD: Obsessive compulsive disorder
OCEBM: Oxford Centre for Evidence-Based Medicine
PET: Positron emission tomography
PMM: Predictive mean matching
PR: Partial response
PRISMA: Preferred Reporting Items for Systematic Reviews and Meta-Analyses
PROSPERO: International Prospective Register of Systematic Reviews
RoB2: Cochrane risk-of-bias tool for randomised trials (Version 2)
ROBINS-I: Risk Of Bias in Non-randomised Studies - of Interventions
SPECT: Single-photon emission computed tomography
STN: Subthalamic Nucleus
Tukey’s HSD: Tukey’s Honestly significant difference
vALIC: Ventral Anterior Limb of Internal Capsule
VC: Ventral Capsule
VS: Ventral Striatum
WHO-QOL-BREF: World Health Organization Quality of Life Brief Version
Y-BOCS: Yale-Brown Obsessive Compulsive Score

## Declarations

### Dissertation statement

This manuscript constitutes the doctoral dissertation of Santhosh G. Thavarajasingam, submitted infulfilment of the requirements for the doctoral degree.
