# Supplementary material for: Predictors of deep brain stimulation response in patients with obsessive compulsive disorder: a systematic review and meta-analysis
Source: Sci Rep. 2026 Jun 4;16:17357. doi: 10.1038/s41598-026-54929-8 (PMC13237133; doi:10.1038/s41598-026-54929-8)
Supplement: Supplementary file 1 — Supplementary Material 1 [file 41598_2026_54929_MOESM1_ESM.docx]

Supplemental Digital Content 1

**Predictors of deep brain stimulation (DBS) response**

**in patients with obsessive compulsive disorder (OCD):**

**a systematic review and meta-analysis**

Santhosh G. Thavarajasingam^1,2,3,4,10,11*^

Sajeenth Vishnu K^4,5^ ; Amir Puyan Divanbeighi Zand^1,3^ ; Daniele S. C. Ramsay^4,5^

Ahmed Salih^4,5^ ; Manuel V. Baby^4,5^ ; Roma D. Thakker^4,5^ ; Jasleen Nagi^4,5^

John Eraifej^1,2,3^ ; Guru Amirthalingam^5^ ; Zoe Shaked^5,6^ ; Hugo Sivov^4,5^

Dragan Jankovic^10,11^ ; Andreas Kramer^10,11^; Denise Linsmayer^8^

Andreas Nowacki^7^ ; Sergiu Groppa^9^ ; Martin B. Glaser^10^

Jan Hinnerk-Mehrkens^11^ ; Florian Ringel^10,11^

Alexander L. Green^1,2,3^

Table of Contents

[Supplementary Table 1 3](#_Toc157511444)

[Supplementary Table 2 6](#_Toc157511445)

[Supplementary Table 3 7](#_Toc157511446)

[Supplementary Table 4 7](#_Toc157511447)

[Supplementary Table 5 8](#_Toc157511448)

[Supplementary Table 6 10](#_Toc157511449)

[Supplementary Table 7 11](#_Toc157511450)

[Supplementary Table 8 12](#_Toc157511451)

[Supplementary Table 9 13](#_Toc157511452)

[Supplementary Table 10 14](#_Toc157511453)

[Supplementary Table 11 15](#_Toc157511454)

[Supplementary Table 12 17](#_Toc157511455)

[Supplementary Table 13 18](#_Toc157511456)

[Supplementary Table 14 21](#_Toc157511457)

[Supplementary Figure 1 26](#_Toc157511458)

[Supplementary Figure 2 28](#_Toc157511459)

[Supplementary Figure 3 29](#_Toc157511460)

[Supplementary Figure 4 30](#_Toc157511461)

[Supplementary Material 1 32](#_Toc157511462)

[References 34](#_Toc157511463)

Supplementary Table 1**: The 27-point checklist furnished by the Preferred Reporting Items for Systematic Reviews and Meta-Analyses (PRISMA) 2020 statement, addressing the individual sections in this systematic review and meta-analysis.**^1^

| **Section and Topic** | **Item #** | **Checklist item** | **Location where item is reported** |
| --- | --- | --- | --- |
| **TITLE** | | |  |
| Title | 1 | Identify the report as a systematic review. | Page 2 |
| **ABSTRACT** | | |  |
| Abstract | 2 | See the PRISMA 2020 for Abstracts checklist. | Page 2 |
| **INTRODUCTION** | | |  |
| Rationale | 3 | Describe the rationale for the review in the context of existing knowledge. | Pages 1-2 |
| Objectives | 4 | Provide an explicit statement of the objective(s) or question(s) the review addresses. | Page 2 |
| **METHODS** | | |  |
| Eligibility criteria | 5 | Specify the inclusion and exclusion criteria for the review and how studies were grouped for the syntheses. | Supplementary Table 3 |
| Information sources | 6 | Specify all databases, registers, websites, organisations, reference lists and other sources searched or consulted to identify studies. Specify the date when each source was last searched or consulted. | Page 2 |
| Search strategy | 7 | Present the full search strategies for all databases, registers and websites, including any filters and limits used. | Supplementary Table 2 |
| Selection process | 8 | Specify the methods used to decide whether a study met the inclusion criteria of the review, including how many reviewers screened each record and each report retrieved, whether they worked independently, and if applicable, details of automation tools used in the process. | Page 2 |
| Data collection process | 9 | Specify the methods used to collect data from reports, including how many reviewers collected data from each report, whether they worked independently, any processes for obtaining or confirming data from study investigators, and if applicable, details of automation tools used in the process. | Page 3 |
| Data items | 10a | List and define all outcomes for which data were sought. Specify whether all results that were compatible with each outcome domain in each study were sought (e.g. for all measures, time points, analyses), and if not, the methods used to decide which results to collect. | Page 3 and Tables 1, 2 |
|  | 10b | List and define all other variables for which data were sought (e.g. participant and intervention characteristics, funding sources). Describe any assumptions made about any missing or unclear information. | Tables 1-3, Supplementary Table 4, Supplementary Material 1 |
| Study risk of bias assessment | 11 | Specify the methods used to assess risk of bias in the included studies, including details of the tool(s) used, how many reviewers assessed each study and whether they worked independently, and if applicable, details of automation tools used in the process. | Page 3 |
| Effect measures | 12 | Specify for each outcome the effect measure(s) (e.g. risk ratio, mean difference) used in the synthesis or presentation of results. | Tables 1 and 2, Figure 2E |
| Synthesis methods | 13a | Describe the processes used to decide which studies were eligible for each synthesis (e.g. tabulating the study intervention characteristics and comparing against the planned groups for each synthesis (item #5)). | Supplementary Table 4 |
|  | 13b | Describe any methods required to prepare the data for presentation or synthesis, such as handling of missing summary statistics, or data conversions. | Page 2-3, Supplementary Material 1 |
|  | 13c | Describe any methods used to tabulate or visually display results of individual studies and syntheses. | Pages 4-7 |
|  | 13d | Describe any methods used to synthesize results and provide a rationale for the choice(s). If meta-analysis was performed, describe the model(s), method(s) to identify the presence and extent of statistical heterogeneity, and software package(s) used. | Page 4 and Supplementary Material 1 |
|  | 13e | Describe any methods used to explore possible causes of heterogeneity among study results (e.g. subgroup analysis, meta-regression). | Page 5 and Figures 2B-2F |
|  | 13f | Describe any sensitivity analyses conducted to assess robustness of the synthesized results. | Page 4 and Supplementary Material 1 |
| Reporting bias assessment | 14 | Describe any methods used to assess risk of bias due to missing results in a synthesis (arising from reporting biases). | Page 3 and Supplementary Figures 1 and 2 |
| Certainty assessment | 15 | Describe any methods used to assess certainty (or confidence) in the body of evidence for an outcome. | Page 3 and Supplementary Table 12 |
| **RESULTS** | | |  |
| Study selection | 16a | Describe the results of the search and selection process, from the number of records identified in the search to the number of studies included in the review, ideally using a flow diagram. | Figure 1A |
|  | 16b | Cite studies that might appear to meet the inclusion criteria, but which were excluded, and explain why they were excluded. | NA |
| Study characteristics | 17 | Cite each included study and present its characteristics. | Table 1 |
| Risk of bias in studies | 18 | Present assessments of risk of bias for each included study. | Page 5 and Supplementary Figures 1 and 2 |
| Results of individual studies | 19 | For all outcomes, present, for each study: (a) summary statistics for each group (where appropriate) and (b) an effect estimate and its precision (e.g. confidence/credible interval), ideally using structured tables or plots. | Table 2 |
| Results of syntheses | 20a | For each synthesis, briefly summarise the characteristics and risk of bias among contributing studies. | Table 1 and Supplementary Figures 1 and 2 |
|  | 20b | Present results of all statistical syntheses conducted. If meta-analysis was done, present for each the summary estimate and its precision (e.g. confidence/credible interval) and measures of statistical heterogeneity. If comparing groups, describe the direction of the effect. | Pages 7-9 , Tables 4 and 5, Figures 3A, 5 and 6, Supplementary Tables 5-11, Supplementary Figures 3 and 4 |
|  | 20c | Present results of all investigations of possible causes of heterogeneity among study results. | Page 13 |
|  | 20d | Present results of all sensitivity analyses conducted to assess the robustness of the synthesized results. | Page 9 |
| Reporting biases | 21 | Present assessments of risk of bias due to missing results (arising from reporting biases) for each synthesis assessed. | Supplementary Figures 1 and 2 |
| Certainty of evidence | 22 | Present assessments of certainty (or confidence) in the body of evidence for each outcome assessed. | Supplementary Table 12 |
| **DISCUSSION** | | |  |
| Discussion | 23a | Provide a general interpretation of the results in the context of other evidence. | Page 10-12 |
|  | 23b | Discuss any limitations of the evidence included in the review. | Page 12 |
|  | 23c | Discuss any limitations of the review processes used. | Page 12 |
|  | 23d | Discuss implications of the results for practice, policy, and future research. | Page 10-14 |
| **OTHER INFORMATION** | | |  |
| Registration and protocol | 24a | Provide registration information for the review, including register name and registration number, or state that the review was not registered. | Page 2 |
|  | 24b | Indicate where the review protocol can be accessed, or state that a protocol was not prepared. | Page 2 |
|  | 24c | Describe and explain any amendments to information provided at registration or in the protocol. | NA |
| Support | 25 | Describe sources of financial or non-financial support for the review, and the role of the funders or sponsors in the review. | Title page/designated section in paper |
| Competing interests | 26 | Declare any competing interests of review authors. | Title page/designated section in paper |
| Availability of data, code and other materials | 27 | Report which of the following are publicly available and where they can be found: template data collection forms; data extracted from included studies; data used for all analyses; analytic code; any other materials used in the review. | Page 46 |

Supplementary Table 2**: Search strategy.**

| **Database** | **Search terms** | **Publication dates** | **Results (n)** |
| --- | --- | --- | --- |
| Medline | ((OCD or obsessive-compulsive disorder or obsessive compulsive disorder) and (DBS or deep brain stimulation)).af. | 1946 - 2022 | n = 1,234 |
| Embase | ((OCD or obsessive-compulsive disorder or obsessive compulsive disorder) and (DBS or deep brain stimulation)).af. | 1946 - 2022 | n = 1,655 |
| Scopus | (TITLE-ABS-  KEY (obsessive AND compulsive AND disorder ) OR TITLE- ABS-KEY ( ocd ) OR TITLE-ABS-KEY ( obsessive- compulsive AND disorder ) AND TITLE-ABS-  KEY (deep AND brain AND stimulation ) AND TITLE-ABS- KEY ( dbs ) ) | 1960 - 2022 | n = 2,090 |
| PubMed | (DBS OR deep brain stimulation) AND (OCD OR obsessive compulsive disorder OR obsessive-compulsive disorder) | 1943 - 2022 | n = 940 |
| JSTOR | Deep brain stimulation AND obsessive-compulsive disorder OR OCD *Journals only | 1944 - 2022 | n = 186 |

Supplementary Table 2 shows the search strategy performed on 10th June 2022, outlining the respective databases, search terms, publication dates chosen as limiting factors, and the number of results from each database.

Supplementary Table 3**: Inclusion and exclusion criteria.**

| **Inclusion criteria** | **Exclusion criteria** |
| --- | --- |
| - Published in the English language - Peer-reviewed journals - Adult human patients who have been diagnosed with obsessive-compulsive disorder - Studies comparing responders to DBS for OCD (positive clinical response) to non-responders (no positive clinical response post DBS) - For meta-analysis: Comparison of patient and disease characteristics of patients who are DBS responders with DBS non-responders (and/or partial responders) or providing comparative data on one of these sub-groups. | - All non-English languages - Commentaries, narrative reviews, letters to editors, books - Any animal studies and lab-based studies - Studies on children and adolescents (<18 years) |

Supplementary Table 3 shows the inclusion and exclusion criteria used to screen the search results.

Supplementary Table 4**: Table of extracted variables.**

| **Extracted variables for qualitative synthesis** | **Extracted variables for quantitative synthesis** | |
| --- | --- | --- |
| - Sample size - Study type and design - Country - Imaging modalities - Definition of full responders, partial responders and non-responders - Main conclusion - Risk of bias | - Age of symptom onset - Illness duration prior to intervention/time of study - Sex - OCD symptoms - Presence of co-morbidities – specifically depression and anxiety - Baseline and post-intervention scores for depression and anxiety - Medications – specifically anti-depressants, anxiolytics, antipsychotics | - DBS duration and follow-up duration - Target site of DBS - Tractography data - DBS settings – voltage, frequency - Complications and side effects from DBS - Baseline Y-BOCS - Improvement in Y-BOCS post intervention - Functional improvement and markers |

Supplementary Table 4 shows the extracted variables in the qualitative synthesis (systematic review) and quantitative synthesis (meta-analysis).

Supplementary Table 5**: Level of evidence of each of the included studies based on the Oxford Centre of Evidence-Based Medicine (OCEBM) Levels of Evidence Tool.**

| **Study number** | **Author, Year** | **Level of evidence** |
| --- | --- | --- |
| 1 | Abelson et al. (2005) | 2 |
| 2 | Acevedo et al. (2023) | 4 |
| 3 | Baldermann et al. (2019) | 3 |
| 4 | Barcia et al. (2019) | 2 |
| 5 | Chabardes et al. (2020) | 3 |
| 6 | Denys et al. (2010) | 2 |
| 7 | Denys et al. (2020)/ Graat et al. (2021) | 3 |
| 8 | Farrand et al. (2018) | 4 |
| 9 | Germann et al. (2022) / Lee et al. (2019) for clinical information | 4 |
| 10 | Graat et al. (2021) | 3 |
| 11 | Graat et al. (2022) | 4 |
| 12 | Greenberg et al. (2006) | 3 |
| 13 | Hartmann et al. (2016) | 3 |
| 14 | Holland et al. (2020) | 3 |
| 15 | Huff et al. (2010) | 2 |
| 16 | Huys et al. (2019) | 3 |
| 17 | Islam et al. (2015) | 4 |
| 18 | Kahn et al. (2021) | 4 |
| 19 | Li et al. (2020) | 2 |
| 20 | Liebrand et al. (2019) | 3 |
| 21 | Liebrand et al. (2021) | 3 |
| 22 | Lopez-Sosa et al. (2021) | 2 |
| 23 | Luyten et al. (2016) | 2 |
| 24 | Mallet et al. (2008/2019) | 2 |
| 25 | Mosley et al. (2021) | 2 |
| 26 | Naesstrom et al. (2021) | 3 |
| 27 | Ooms et al. (2014) | 2 |
| 28 | Parvaresh-Rizi et al. (2022) | 4 |
| 29 | Raymaekers et al. (2017) | 3 |
| 30 | Sildatke et al. (2021) | 2 |
| 31 | Tsai et al. (2012) | 2 |
| 32 | Tsai et al. (2014) | 4 |
| 33 | Tyagi et al. (2019) | 2 |
| 34 | vanderVlis et al. (2021) | 3 |
| 35 | Welter et al. (2011) | 3 |
| 36 | Widge et al. (2022) | 4 |
| 37 | Winter et al. (2021) | 4 |
| 38 | Voon et al. (2018) | 2 |

Supplementary Table 5 shows the results of the analysis of the strength/quality of scientific evidence of all included studies using the Oxford Centre of Evidence-Based Medicine (OCEBM) Levels of Evidence tool.^2^

Supplementary Table 6**: Chi-squared analysis of categorical clinical predictors of DBS between Responders, Partial Responders and Non-Responders.**

| **Variable** | **X_Squared** | **P_Value** |
| --- | --- | --- |
| Tractography | 0.628357 | 0.730389 |
| ALIC | 0.120205 | 0.941668 |
| NAcc_Striatal_axis | 1.31331 | 0.518583 |
| BNST | 0.39021 | 0.822748 |
| VC_VS | 0.583067 | 0.747117 |
| NAcc_vALIC | 3.355075 | 0.186833 |

Supplementary Table 6 presents the results of the Chi-squared tests run on each categorical clinical predictor for OCD patients, stratified into DBS Responders, Partial Responders, and Non-Responders (unimputed data). The table lists each predictor along with the corresponding Chi-squared statistic and p-value. Asterisks (*) denote predictors with significant differences (p < 0.05) between the response status groups. No categorical clinical predictors reached statistical significance (p < 0.05). The abbreviations used in the table are as follows: Anterior Limb of Internal Capsule (ALIC), Bed Nucleus of Stria Terminalis (BNST), Nucleus Accumbens (NAcc), Subthalamic Nucleus (STN), Ventral Anterior Limb of Internal Capsule (vALIC), Ventral Capsule (VC), Ventral Striatum (VS).

Supplementary Table 7**: ANOVA analysis of continuous clinical predictors of DBS between Responders, Partial Responders and Non-Responders.**

| **Variable** | **Sum_Sq** | **Mean_Sq** | **F_Value** | **P_Value** |
| --- | --- | --- | --- | --- |
| Age | 51.46675 | 25.73337 | 0.457185 | 0.635325 |
| Females | 0.176054 | 0.088027 | 0.711326 | 0.495535 |
| Illness Duration | 14.83392 | 7.416958 | 0.08115 | 0.922203 |
| Onset Age | 201.9293 | 100.9647 | 1.755013 | 0.18663 |
| Pre-Surgery Duration | 156.9481 | 78.47404 | 0.768391 | 0.472389 |
| Major Depression Disorder | 0.008598 | 0.004299 | 0.030819 | 0.969678 |
| Baseline Y-BOCS | 25.31248 | 12.65624 | 1.699718 | 0.192544 |
| Antidepressants | 0.527604 | 0.263802 | 3.425189 | 0.047239 * |
| Anxiolytics | 0.393034 | 0.196517 | 1.692375 | 0.204497 |
| Antipsychotics | 0.321951 | 0.160976 | 1.411701 | 0.261149 |
| Contamination/Cleaning (Baseline) | 0.117562 | 0.058781 | 0.49505 | 0.614277 |
| Harm/Checking (Baseline) | 0.121459 | 0.060729 | 0.55247 | 0.581271 |
| Aggression/Intrusive Thoughts (Baseline) | 0.05187 | 0.025935 | 0.24036 | 0.787951 |
| Symmetry/Hoarding/Perfectionism (Baseline) | 0.248383 | 0.124191 | 1.221561 | 0.308148 |
| Mental/Somatic/Spiritual Rituals (Baseline) | 0.462668 | 0.231334 | 2.740999 | 0.080667 |
| Anxiety/Avoidance/Fear (Baseline) | 0.268368 | 0.134184 | 1.432798 | 0.255036 |
| Baseline Depression (HAM-D) | 166.7296 | 83.36479 | 1.747791 | 0.204035 |
| Baseline GAF | 31.93451 | 15.96725 | 0.261387 | 0.773021 |
| Neurological Side Effects | 0.317927 | 0.158964 | 1.243195 | 0.31089 |
| Psychiatric Side Effects | 0.314302 | 0.157151 | 1.915991 | 0.177667 |
| Elevated Mood/Hypomania | 0.174638 | 0.087319 | 1.348116 | 0.282333 |

Supplementary Table 7 features the results of one-way ANOVA analyses performed independently on each continuous clinical predictor for OCD patients, stratified by DBS response status (unimputed data). Detailed information for each predictor includes the sum of squares, mean square, F-value, and associated p-value. Predictors yielding a significant F statistic (p < 0.05) are marked with an asterisk (*) and were further subjected to Tukey's HSD post-hoc tests

Supplementary Table 8**: Post-Hoc Tukey's HSD Analysis of significant clinical predictors of DBS response (p < 0.05)**

| **Variable** | **Term** | **Contrast** | **Estimate** | **Conf.low** | **Conf.high** | **Adj.p.value** |
| --- | --- | --- | --- | --- | --- | --- |
| Antidepressants | Response_Status | Partial Responder-Non-Responder | 0.020952 | -0.3063 | 0.348205 | 0.98621 |
| Antidepressants | Response_Status | Responder-Non-Responder | -0.26697 | -0.5542 | 0.020256 | 0.072243 |
| Antidepressants | Response_Status | Responder-Partial Responder | -0.28792 | -0.62061 | 0.044766 | 0.099425 |

Supplementary Table 8 delineates the post-hoc Tukey's HSD results for predictors found significant in the ANOVA (unimputed data). Each row provides detailed pairwise comparisons, enumerating estimated mean difference, 95% confidence intervals and the associated p-value. For each predictor, tests were run independently, with each response status acting as a reference category in turn. An asterisk (*) denotes statistical significance (p < 0.05).

Supplementary Table 9**: Univariate linear regression analyses of continuous outcome variables of DBS response (p < 0.05) and stepwise built multivariate linear regression models using the unimputed dataset.**

| **Explanatory Variable** | **Outcome Variable** | **Estimate (Beta)** | **SE** | **t-statistic** | **p-value** | **Model** |
| --- | --- | --- | --- | --- | --- | --- |
| Anxiolytics | Post_Y_BOCS_short_term | -16.399944 | 4.010263 | -4.089493 | 0.000855 *** | Univariate |
| Anxiolytics | Improvement_short_term | 0.533875 | 0.128182 | 4.164989 | 0.000953 *** | Univariate |
| Symmetry_Hoarding_Perfectionism_Baseline | Improvement_short_term | -0.352085 | 0.143068 | -2.460968 | 0.031624 * | Univariate |
| Baseline_Y_BOCS | Improvement_long_term | 0.033423 | 0.011413 | 2.928508 | 0.005117 ** | Univariate |
| Anxiolytics | Post_Y_BOCS_short_term | -16.3999 | 4.010263 | -4.08949 | 0.000855 *** | Stepwise multivariate |
| Anxiolytics | Improvement_short_term | 0.547204 | 0.178417 | 3.06699 | 0.018144 * | Stepwise multivariate |
| Baseline_Y_BOCS | Improvement_long_term | 0.033423 | 0.011413 | 2.928508 | 0.005117 ** | Stepwise multivariate |

Supplementary Table 9 presents the univariate and stepwise built multivariate linear regression analyses examining the relationship between selected explanatory variables and various continuous outcome measures related to DBS response. Single asterisks (*) indicate p-values less than 0.05, while double asterisks (**) denote p-values less than 0.01, signifying statistical significance. Univariate regression outcomes are marked with a caret (^). The table delineates the regression coefficient (Estimate), standard error (SE), t-statistic, adjusted R-squared value, and p-value for each relationship studied. The explanatory variables were independently assessed for their impact on the short-term Y-BOCS scores, with the significance of their associations denoted accordingly. The stepwise selection process in the multivariate analysis incorporated these variables from the univariate analyses into final models to predict short-term and long-term Y-BOCS scores and the improvement over these periods if their p < 0.05. Anxiolytic use was significantly associated with lower short-term Y-BOCS scores and positively correlated with short-term improvement, suggesting a reduction in symptom burden. Conversely, baseline traits of symmetry, hoarding, and perfectionism were negatively associated with short-term improvement. A higher baseline Y-BOCS score was positively associated with long-term improvement in both univariate and stepwise multivariate analyses, emerging as the most consistent predictor. The final multivariate model also retained baseline Y-BOCS as a significant explanatory variable for long-term improvement.

Supplementary Table 10**: Univariate logistic regression analysis of continuous outcome variables of DBS response (p < 0.05) using the unimputed dataset.**

| **Explanatory Variable** | **Outcome Variable** | **Estimate (Beta)** | **SE** | **z-statistic** | **p-value** |
| --- | --- | --- | --- | --- | --- |
| Antidepressant | Responder | -3.95212 | 1.787526 | -2.21094 | 0.02704 * |

Supplementary Table 10 delineates the univariate logistic regression analysis performed to explore the predictive value of selected explanatory variables on the binary outcome of DBS responders. The table presents only one statistically significant model, with an asterisk (*) marking the p-values less than 0.05, indicating statistical significance. The analysis delineates the regression coefficient (Estimate), standard error (SE), z-statistic and p-value of the identified relationship, showcasing its strength and precision. Specifically, the use of Antidepressants emerged as a significant predictor in the univariate logistic regression, with a negative beta value suggesting a decrease in the likelihood of being a responder to DBS when Antidepressants are used. However, given the absence of other significant univariate relationships, a stepwise multivariate linear regression model was not produced. The results underscore the complexity of DBS response predictors and highlight the necessity for a broader analysis to uncover the multifaceted nature of treatment outcomes.

Supplementary Table 11**: Univariate linear regression analyses of continuous outcome variables of DBS response (p < 0.05) and stepwise built multivariate linear regression models using the PMM-imputed dataset.**

| **Explanatory Variable** | **Outcome Variable** | **Estimate (Beta)** | **SE** | **t-statistic** | **p-value** | **Model** |
| --- | --- | --- | --- | --- | --- | --- |
| Baseline_Y_BOCS | Post_Y_BOCS_short_term | 0.7550798 | 0.3491417 | 2.1626742 | 0.0341395 * | Univariate |
| Onset_Age | Post_Y_BOCS_long_term | 0.1782698 | 0.0862244 | 2.0675090 | 0.0425518 * | Univariate |
| Antipsychotics | Post_Y_BOCS_long_term | 7.7220808 | 2.2869475 | 3.3765885 | 0.0012254 ** | Univariate |
| Antipsychotics | Improvement_short_term | -0.2084612 | 0.0969678 | -2.1497972 | 0.0351853 * | Univariate |
| Onset_Age | Improvement_long_term | -0.0072219 | 0.0030205 | -2.3909421 | 0.0196215 * | Univariate |
| Baseline_Y_BOCS | Improvement_long_term | 0.0283167 | 0.0108295 | 2.6147791 | 0.0110189 * | Univariate |
| Antipsychotics | Improvement_long_term | -0.1915751 | 0.0843487 | -2.2712285 | 0.0263513 * | Univariate |
| Contamination_Cleaning_Baseline | Improvement_long_term | 0.0032152 | 0.0014774 | 2.1762586 | 0.0330657 * | Univariate |
| Baseline_Y_BOCS | Post_Y_BOCS_short_term | 0.75508 | 0.349142 | 2.162674 | 0.034139 * | Stepwise built multivariate |
| Onset_Age | Post_Y_BOCS_long_term | 0.132395 | 0.08263 | 1.602271 | 0.113871 | Stepwise built multivariate |
| Antipsychotics | Post_Y_BOCS_long_term | 7.056001 | 2.298564 | 3.069743 | 0.003108* | Stepwise built multivariate |
| Antipsychotics | Improvement_short_term | -0.20846 | 0.096968 | -2.1498 | 0.035185* | Stepwise built multivariate |
| Onset_Age | Improvement_long_term | -0.00689 | 0.002825 | -2.43705 | 0.017598* | Stepwise built multivariate |
| Baseline_Y_BOCS | Improvement_long_term | 0.024661 | 0.010234 | 2.409694 | 0.018852* | Stepwise built multivariate |
| Antipsychotics | Improvement_long_term | -0.13341 | 0.078881 | -1.69131 | 0.095642 | Stepwise built multivariate |
| Contamination_Cleaning_Baseline | Improvement_long_term | 0.002676 | 0.001365 | 1.960858 | 0.05425 | Stepwise built multivariate |

Supplementary Table 11 presents the univariate linear regression analysis examining the relationship between selected explanatory variables and various continuous outcome measures related to DBS response with a predictive mean matching (PMM) imputed dataset. Single asterisks (*) indicate p-values less than 0.05, while double asterisks (**) denote p-values less than 0.01 and triple asterisks (***) p-values less than 0.001, signifying increasing levels of statistical significance. A higher baseline Y-BOCS score was significantly associated with both lower short-term Y-BOCS scores and greater long-term improvement, indicating its predictive value for favorable DBS outcomes. In contrast, baseline use of antipsychotics was significantly negatively associated with short- and long-term improvement (p<0.05). Furthermore, contamination/cleaning symptoms at baseline statistically significant negative association with long-term improvement (p<0.05).

Supplementary Table 12**: Univariate logistic regressions of categorical outcome variables of DBS response (p < 0.05) and stepwise built multivariate logistic regression models (PMM data).**

| **Explanatory variable** | **Estimate (Beta)** | **SE** | **z-statistic** | **p-value** | **Outcome variable** | **Model** |
| --- | --- | --- | --- | --- | --- | --- |
| Antipsychotics | -1.5135 | 0.7348 | -2.06 | 0.0394 * | Responder | Univariate |
| Anxiety_Avoidance_Fear_Baseline | 1.641529 | 0.709295 | 2.31431 | 0.020650733 * | Partial_Responder | Univariate |
| Antipsychotics | 1.665358 | 0.770736 | 2.160736 | 0.030715733 * | Non_Responder | Univariate |
| Antipsychotics | -1.5135 | 0.734837 | -2.059637319 | 0.039433226* | Responder | Stepwise built multivariate |
| Anxiety_Avoidance_Fear_Baseline | 1.641529 | 0.709295 | 2.314309832 | 0.020650733* | Partial_Responder | Stepwise built multivariate |
| Antipsychotics | 1.665358 | 0.770736 | 2.160736074 | 0.030715733* | Non_Responder | Stepwise built multivariate |

Supplementary Table 12 shows the univariate logistic regression analyses of the imputed dataset, which found that antipsychotic use was negatively associated with being a responder (p < 0.05), while baseline anxiety/avoidance/fear symptoms were positively associated with being a partial responder (p < 0.05). Antipsychotic use was also positively associated with non-response (p < 0.05).

Supplementary Table 13**: Final mixed-effects multivariate linear regression model of continuous outcome variables of DBS response (PMM) including target locations.**

| **Explanatory variable** | **Outcome variable** | **Estimate** | **SE** | **t-statistic** | **p-value** |
| --- | --- | --- | --- | --- | --- |
| Baseline_Y_BOCS | Responder | 0.022203 | 0.025576 | 0.868124 | 0.389474 |
| Antidepressants | Responder | -0.17932 | 0.180107 | -0.99564 | 0.324219 |
| Antipsychotics | Responder | -0.5177 | 0.20376 | -2.54074 | 0.014213 * |
| Aggression_Intrusive_Thoughts_Baseline | Responder | 0.416431 | 0.186015 | 2.238693 | 0.029657 * |
| Symmetry_Hoarding_Perfectionism_Baseline | Responder | -0.21381 | 0.17239 | -1.24025 | 0.220672 |
| Anxiety_Avoidance_Fear_Baseline | Responder | -0.28686 | 0.183076 | -1.56691 | 0.123443 |
| Elevated_Mood_Hypomania | Responder | 0.031635 | 0.254602 | 0.124253 | 0.901613 |
| Tractography1 | Responder | 0.351506 | 0.139642 | 2.5172 | 0.015081* |
| ALIC1 | Responder | -0.24743 | 0.157798 | -1.56804 | 0.123179 |
| NAcc_Striatal_axis1 | Responder | -0.22977 | 0.339424 | -0.67694 | 0.501563 |
| BNST1 | Responder | -0.01353 | 0.166868 | -0.08106 | 0.935715 |
| Anterior_hypothalamus1 | Responder | 0.655761 | 0.548966 | 1.194538 | 0.237904 |
| MFB1 | Responder | 0.371525 | 0.409292 | 0.907725 | 0.368378 |
| vALIC1 | Responder | -0.33057 | 0.322035 | -1.02651 | 0.30959 |
| Nacc1 | Responder | -0.30078 | 0.152223 | -1.97592 | 0.053696 |
| VC_VS1 | Responder | 0.159875 | 0.194581 | 0.821637 | 0.415184 |
| NAcc_vALIC1 | Responder | 0.737673 | 0.371786 | 1.98413 | 0.052744 |
| Baseline_Y_BOCS | Partial_Responder | 0.00484 | 0.022836 | 0.211951 | 0.833007 |
| Antidepressants | Partial_Responder | -0.10258 | 0.160811 | -0.63789 | 0.526454 |
| Antipsychotics | Partial_Responder | -0.05262 | 0.18193 | -0.28926 | 0.77358 |
| Aggression_Intrusive_Thoughts_Baseline | Partial_Responder | -0.11535 | 0.166086 | -0.6945 | 0.490583 |
| Symmetry_Hoarding_Perfectionism_Baseline | Partial_Responder | 0.164844 | 0.153921 | 1.070967 | 0.289327 |
| Anxiety_Avoidance_Fear_Baseline | Partial_Responder | 0.419106 | 0.163462 | 2.563942 | 0.013401 * |
| Elevated_Mood_Hypomania | Partial_Responder | 0.342818 | 0.227325 | 1.508055 | 0.137834 |
| Tractography1 | Partial_Responder | -0.15357 | 0.124681 | -1.23174 | 0.223807 |
| ALIC1 | Partial_Responder | 0.009543 | 0.140892 | 0.067733 | 0.946268 |
| NAcc_Striatal_axis1 | Partial_Responder | 0.1975 | 0.303059 | 0.651687 | 0.517588 |
| BNST1 | Partial_Responder | 0.221473 | 0.148991 | 1.486487 | 0.143429 |
| Anterior_hypothalamus1 | Partial_Responder | -0.47629 | 0.490151 | -0.97173 | 0.335864 |
| MFB1 | Partial_Responder | 0.201743 | 0.365442 | 0.552052 | 0.583372 |
| vALIC1 | Partial_Responder | 0.074393 | 0.287533 | 0.258727 | 0.796908 |
| Nacc1 | Partial_Responder | 0.257706 | 0.135914 | 1.896091 | 0.063734 |
| VC_VS1 | Partial_Responder | -0.03316 | 0.173734 | -0.19087 | 0.849401 |
| NAcc_vALIC1 | Partial_Responder | 0.120799 | 0.331954 | 0.363901 | 0.717466 |
| Baseline_Y_BOCS | Non_Responder | -0.02704 | 0.024367 | -1.10986 | 0.272368 |
| Antidepressants | Non_Responder | 0.281903 | 0.171589 | 1.642897 | 0.106679 |
| Antipsychotics | Non_Responder | 0.570325 | 0.194123 | 2.937963 | 0.004986 ** |
| Aggression_Intrusive_Thoughts_Baseline | Non_Responder | -0.30108 | 0.177217 | -1.69896 | 0.095542 |
| Symmetry_Hoarding_Perfectionism_Baseline | Non_Responder | 0.048963 | 0.164236 | 0.298122 | 0.766845 |
| Anxiety_Avoidance_Fear_Baseline | Non_Responder | -0.13224 | 0.174417 | -0.7582 | 0.451891 |
| Elevated_Mood_Hypomania | Non_Responder | -0.37445 | 0.24256 | -1.54376 | 0.128954 |
| Tractography1 | Non_Responder | -0.19793 | 0.133037 | -1.48779 | 0.143086 |
| ALIC1 | Non_Responder | 0.237891 | 0.150335 | 1.582412 | 0.119861 |
| NAcc_Striatal_axis1 | Non_Responder | 0.032271 | 0.32337 | 0.099795 | 0.920906 |
| BNST1 | Non_Responder | -0.20795 | 0.158976 | -1.30803 | 0.196844 |
| Anterior_hypothalamus1 | Non_Responder | -0.17947 | 0.523001 | -0.34315 | 0.732925 |
| MFB1 | Non_Responder | -0.57327 | 0.389934 | -1.47017 | 0.147781 |
| vALIC1 | Non_Responder | 0.256181 | 0.306803 | 0.835001 | 0.407689 |
| Nacc1 | Non_Responder | 0.043074 | 0.145023 | 0.297014 | 0.767686 |
| VC_VS1 | Non_Responder | -0.12671 | 0.185377 | -0.68355 | 0.497417 |
| NAcc_vALIC1 | Non_Responder | -0.85847 | 0.354202 | -2.42368 | 0.019025 * |

In Table 13, the results of the final mixed-effects multivariate linear regression model of categorical outcome variables of DBS response using the predictive mean matching (PMM) imputed dataset including target locations is shown. Single asterisks (*) indicate p-values less than 0.05, while double asterisks (**) denote p-values less than 0.01, signifying statistical significance. The table delineates the regression coefficient (Estimate), standard error (SE), t-statistic, and p-value for each relationship studied.

Supplementary Table 14**: Final mixed-effects multivariate logistic regression model of categorical outcome variables of DBS response (PMM) including target locations.**

| **Explanatory variable** | **Outcome variable** | **Estimate** | **SE** | **z-statistic** | **p-value** |
| --- | --- | --- | --- | --- | --- |
| Baseline_Y_BOCS | Responder | 0.223926 | 0.162854 | 1.375011 | 0.169128 |
| Antidepressants | Responder | -1.54474 | 1.198681 | -1.2887 | 0.197504 |
| Antipsychotics | Responder | -3.50699 | 1.382744 | -2.53625 | 0.011205 * |
| Aggression_Intrusive_Thoughts_Baseline | Responder | 2.992882 | 1.244398 | 2.405085 | 0.016169 * |
| Symmetry_Hoarding_Perfectionism_Baseline | Responder | -2.03051 | 1.148837 | -1.76745 | 0.077153 |
| Anxiety_Avoidance_Fear_Baseline | Responder | -1.87827 | 1.054365 | -1.78142 | 0.074844 |
| Elevated_Mood_Hypomania | Responder | 0.021484 | 1.514194 | 0.014189 | 0.988679 |
| Tractography1 | Responder | 2.662483 | 1.022546 | 2.603777 | 0.00922 ** |
| ALIC1 | Responder | -1.69149 | 1.032389 | -1.63842 | 0.101334 |
| NAcc_Striatal_axis1 | Responder | -1.77481 | 2.125192 | -0.83513 | 0.403645 |
| BNST1 | Responder | -0.05858 | 0.962016 | -0.0609 | 0.951442 |
| Anterior_hypothalamus1 | Responder | 18.54343 | 3956.181 | 0.004687 | 0.99626 |
| MFB1 | Responder | 15.9636 | 2261.961 | 0.007057 | 0.994369 |
| vALIC1 | Responder | -16.4815 | 2261.96 | -0.00729 | 0.994186 |
| Nacc1 | Responder | -1.88161 | 0.943491 | -1.99431 | 0.046119 * |
| VC_VS1 | Responder | 1.183318 | 1.06883 | 1.107115 | 0.268244 |
| NAcc_vALIC1 | Responder | 19.51173 | 2261.96 | 0.008626 | 0.993118 |
| Baseline_Y_BOCS | Partial_Responder | -0.00626 | 0.174158 | -0.03596 | 0.971311 |
| Antidepressants | Partial_Responder | -0.91987 | 1.143158 | -0.80468 | 0.421006 |
| Antipsychotics | Partial_Responder | -0.47212 | 1.446484 | -0.32639 | 0.744127 |
| Aggression_Intrusive_Thoughts_Baseline | Partial_Responder | -0.59524 | 1.346991 | -0.44191 | 0.658558 |
| Symmetry_Hoarding_Perfectionism_Baseline | Partial_Responder | 1.252684 | 1.183235 | 1.058694 | 0.289739 |
| Anxiety_Avoidance_Fear_Baseline | Partial_Responder | 3.214315 | 1.46791 | 2.189722 | 0.028544 * |
| Elevated_Mood_Hypomania | Partial_Responder | 2.288662 | 1.646514 | 1.390004 | 0.164528 |
| Tractography1 | Partial_Responder | -0.94574 | 1.04992 | -0.90077 | 0.367709 |
| ALIC1 | Partial_Responder | 0.493485 | 1.128591 | 0.437257 | 0.661925 |
| NAcc_Striatal_axis1 | Partial_Responder | -13.3343 | 3670.377 | -0.00363 | 0.997101 |
| BNST1 | Partial_Responder | 1.837248 | 1.388967 | 1.322744 | 0.18592 |
| Anterior_hypothalamus1 | Partial_Responder | -18.6239 | 6522.639 | -0.00286 | 0.997722 |
| MFB1 | Partial_Responder | 17.08988 | 3407.509 | 0.005015 | 0.995998 |
| vALIC1 | Partial_Responder | -14.6916 | 3407.509 | -0.00431 | 0.99656 |
| Nacc1 | Partial_Responder | 2.390613 | 1.186192 | 2.015367 | 0.043866 * |
| VC_VS1 | Partial_Responder | 0.793061 | 1.398667 | 0.567012 | 0.570706 |
| NAcc_vALIC1 | Partial_Responder | 16.46748 | 3407.509 | 0.004833 | 0.996144 |
| Baseline_Y_BOCS | Non_Responder | -0.16379 | 0.163341 | -1.00273 | 0.31599 |
| Antidepressants | Non_Responder | 2.934642 | 1.627899 | 1.802717 | 0.071433 |
| Antipsychotics | Improvement_short_term | -0.16492 | 0.105029 | -1.57023 | 0.121534 |
| Aggression_Intrusive_Thoughts_Baseline | Improvement_short_term | 0.02058 | 0.101425 | 0.202906 | 0.839884 |
| Symmetry_Hoarding_Perfectionism_Baseline | Improvement_short_term | -0.0087 | 0.095728 | -0.09089 | 0.927879 |
| Anxiety_Avoidance_Fear_Baseline | Improvement_short_term | -0.10259 | 0.100019 | -1.02575 | 0.309059 |
| Elevated_Mood_Hypomania | Improvement_short_term | 0.240401 | 0.146834 | 1.637232 | 0.106731 |
| Baseline_Y_BOCS | Improvement_long_term | 0.033298 | 0.01204 | 2.765707 | 0.007505 ** |
| Antidepressants | Improvement_long_term | -0.05673 | 0.083743 | -0.67738 | 0.500726 |
| Antipsychotics | Improvement_long_term | -0.12312 | 0.087671 | -1.40432 | 0.165296 |
| Aggression_Intrusive_Thoughts_Baseline | Improvement_long_term | 0.004462 | 0.084663 | 0.052698 | 0.958145 |
| Symmetry_Hoarding_Perfectionism_Baseline | Improvement_long_term | -0.06391 | 0.079907 | -0.79984 | 0.426909 |
| Anxiety_Avoidance_Fear_Baseline | Improvement_long_term | -0.09442 | 0.083489 | -1.13091 | 0.262521 |
| Elevated_Mood_Hypomania | Improvement_long_term | 0.155085 | 0.122567 | 1.265309 | 0.210573 |

In Table 14, the results of the final mixed-effects multivariate logistic regression model of continuous outcome variables of DBS response using the predictive mean matching (PMM) imputed dataset including target locations is shown. Single asterisks (*) indicate p-values less than 0.05, while double asterisks (**) denote p-values less than 0.01, signifying statistical significance. The table delineates the regression coefficient (Estimate), standard error (SE), z-statistic, and p-value for each relationship studied.

Supplementary Table 15**: Final mixed-effects multivariate linear regression model of continuous outcome variables of DBS response (PMM) excluding high risk of bias studies**

| **Explanatory variable** | **Outcome variable** | **Estimate** | **SE** | **t-statistic** | **p-value** |
| --- | --- | --- | --- | --- | --- |
| Baseline_Y_BOCS | Post_Y_BOCS_short_term | 0.735214 | 0.436715 | 1.683509 | 0.099052 |
| Antidepressants | Post_Y_BOCS_short_term | -5.31038 | 3.281302 | -1.61837 | 0.112419 |
| Antipsychotics | Post_Y_BOCS_short_term | 2.922961 | 3.473993 | 0.841384 | 0.404486 |
| Aggression_Intrusive_Thoughts_Baseline | Post_Y_BOCS_short_term | 2.478438 | 3.388391 | 0.73145 | 0.468214 |
| Symmetry_Hoarding_Perfectionism_Baseline | Post_Y_BOCS_short_term | -2.65245 | 2.893609 | -0.91666 | 0.364102 |
| Anxiety_Avoidance_Fear_Baseline | Post_Y_BOCS_short_term | 0.976311 | 3.307553 | 0.295176 | 0.769188 |
| Elevated_Mood_Hypomania | Post_Y_BOCS_short_term | -8.24012 | 4.604581 | -1.78955 | 0.080111 |
| Baseline_Y_BOCS | Post_Y_BOCS_long_term | -0.32417 | 0.379991 | -0.85309 | 0.398029 |
| Antidepressants | Post_Y_BOCS_long_term | 0.485764 | 2.855102 | 0.170139 | 0.865647 |
| Antipsychotics | Post_Y_BOCS_long_term | 5.287838 | 3.022765 | 1.749338 | 0.086902 |
| Aggression_Intrusive_Thoughts_Baseline | Post_Y_BOCS_long_term | -0.49281 | 2.948281 | -0.16715 | 0.867984 |
| Symmetry_Hoarding_Perfectionism_Baseline | Post_Y_BOCS_long_term | 1.065195 | 2.517766 | 0.423071 | 0.674215 |
| Anxiety_Avoidance_Fear_Baseline | Post_Y_BOCS_long_term | 3.922684 | 2.877944 | 1.363016 | 0.179512 |
| Elevated_Mood_Hypomania | Post_Y_BOCS_long_term | -5.69266 | 4.006504 | -1.42086 | 0.162102 |
| Baseline_Y_BOCS | Improvement_short_term | 0.024732 | 0.015509 | 1.594641 | 0.117641 |
| Antidepressants | Improvement_short_term | -0.04053 | 0.116531 | -0.34779 | 0.729582 |
| Antipsychotics | Improvement_short_term | -0.22448 | 0.123374 | -1.81952 | 0.075344 |
| Aggression_Intrusive_Thoughts_Baseline | Improvement_short_term | -0.01683 | 0.120334 | -0.13988 | 0.889369 |
| Symmetry_Hoarding_Perfectionism_Baseline | Improvement_short_term | -0.02577 | 0.102762 | -0.2508 | 0.803082 |
| Anxiety_Avoidance_Fear_Baseline | Improvement_short_term | -0.0522 | 0.117463 | -0.44443 | 0.658813 |
| Elevated_Mood_Hypomania | Improvement_short_term | 0.279137 | 0.163525 | 1.707 | 0.094565 |
| Baseline_Y_BOCS | Improvement_long_term | 0.035737 | 0.013849 | 2.580431 | 0.013125* |
| Antidepressants | Improvement_long_term | -0.038 | 0.104056 | -0.3652 | 0.716635 |
| Antipsychotics | Improvement_long_term | -0.08587 | 0.110167 | -0.77943 | 0.439721 |
| Aggression_Intrusive_Thoughts_Baseline | Improvement_long_term | 0.017816 | 0.107452 | 0.165806 | 0.869036 |
| Symmetry_Hoarding_Perfectionism_Baseline | Improvement_long_term | -0.04265 | 0.091762 | -0.46477 | 0.64429 |
| Anxiety_Avoidance_Fear_Baseline | Improvement_long_term | -0.12752 | 0.104889 | -1.21576 | 0.230278 |
| Elevated_Mood_Hypomania | Improvement_long_term | 0.159104 | 0.14602 | 1.089606 | 0.281562 |

In Table 15, the results of the final mixed-effects multivariate linear regression model of continuous outcome variables of DBS response using the predictive mean matching (PMM) imputed dataset including target locations is shown. Single asterisks (*) indicate p-values less than 0.05, while double asterisks (**) denote p-values less than 0.01, signifying statistical significance. The table delineates the regression coefficient (Estimate), standard error (SE), t-statistic, and p-value for each relationship studied.

Supplementary Table 16**: Final mixed-effects multivariate logistic regression model of categorical outcome variables of DBS response (PMM) excluding high risk of bias studies**

| **Explanatory variable** | **Outcome variable** | **Estimate** | **SE** | **z-statistic** | **p-value** |
| --- | --- | --- | --- | --- | --- |
| Baseline_Y_BOCS | Responder | 0.324248 | 0.148129 | 2.188957 | 0.0286* |
| Antidepressants | Responder | -0.19277 | 0.108181 | -1.7819 | 0.074765 |
| Antipsychotics | Responder | -0.03242 | 0.054733 | -0.59232 | 0.553635 |
| Aggression_Intrusive_Thoughts_Baseline | Responder | 1.861575 | 1.044437 | 1.782373 | 0.074688 |
| Symmetry_Hoarding_Perfectionism_Baseline | Responder | -0.1481 | 0.83157 | -0.1781 | 0.858646 |
| Anxiety_Avoidance_Fear_Baseline | Responder | -1.35807 | 0.952931 | -1.42515 | 0.154113 |
| Elevated_Mood_Hypomania | Responder | 0.338207 | 1.265768 | 0.267195 | 0.789319 |
| Baseline_Y_BOCS | Partial_Responder | -0.17565 | 0.152401 | -1.15258 | 0.249084 |
| Antidepressants | Partial_Responder | 0.219219 | 0.131394 | 1.66841 | 0.095234 |
| Antipsychotics | Partial_Responder | -0.09143 | 0.063947 | -1.42972 | 0.152798 |
| Aggression_Intrusive_Thoughts_Baseline | Partial_Responder | -2.20987 | 1.248878 | -1.76949 | 0.076813 |
| Symmetry_Hoarding_Perfectionism_Baseline | Partial_Responder | 0.678517 | 0.945289 | 0.717788 | 0.472888 |
| Anxiety_Avoidance_Fear_Baseline | Partial_Responder | 2.531137 | 1.122061 | 2.255793 | 0.024084* |
| Elevated_Mood_Hypomania | Partial_Responder | 1.174609 | 1.494774 | 0.78581 | 0.431979 |
| Baseline_Y_BOCS | Non_Responder | -0.13157 | 0.123324 | -1.06686 | 0.286037 |
| Antidepressants | Non_Responder | 0.03413 | 0.108775 | 0.313763 | 0.753701 |
| Antipsychotics | Non_Responder | 0.126112 | 0.065887 | 1.914074 | 0.055611 |
| Aggression_Intrusive_Thoughts_Baseline | Non_Responder | -0.12546 | 1.028076 | -0.12203 | 0.902875 |
| Symmetry_Hoarding_Perfectionism_Baseline | Non_Responder | -0.56332 | 0.846394 | -0.66556 | 0.505695 |
| Anxiety_Avoidance_Fear_Baseline | Non_Responder | -0.77018 | 0.953644 | -0.80762 | 0.419309 |
| Elevated_Mood_Hypomania | Non_Responder | -1.23801 | 1.341036 | -0.92317 | 0.355917 |

In Table 14, the results of the final mixed-effects multivariate logistic regression model of categorical outcome variables of DBS response using the predictive mean matching (PMM) imputed dataset including target locations is shown. Single asterisks (*) indicate p-values less than 0.05, while double asterisks (**) denote p-values less than 0.01, signifying statistical significance. The table delineates the regression coefficient (Estimate), standard error (SE), z-statistic, and p-value for each relationship studied.

Supplementary Figure 1**: Risk of bias analysis of all included non-randomised studies (ROBINS-I tool).**


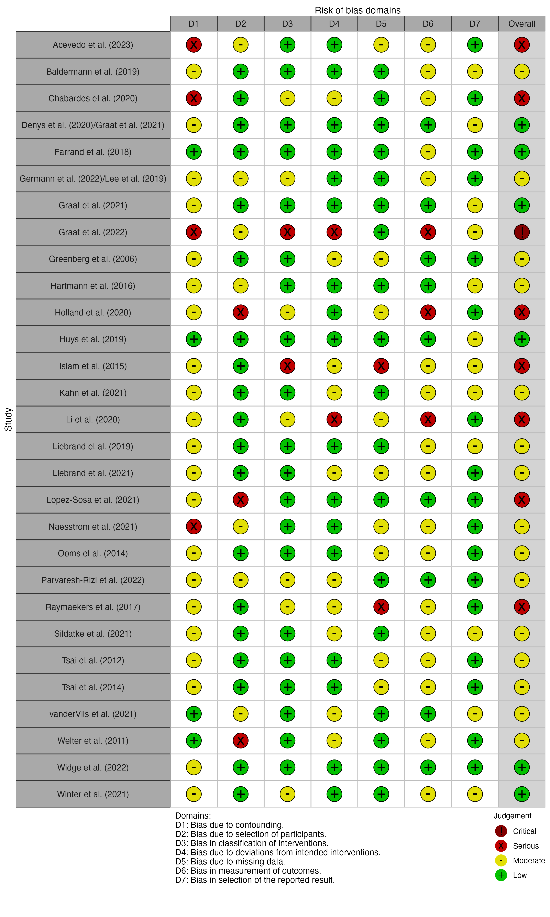


Supplementary Figure 1 shows the results of the risk of bias analysis of all included non-randomised studies using the ROBINS-I tool^3^: Chabardes et al.^4^, Holland et al.^5^, Islam et al.^6^, Li et al.^7^, Lopez-Sosa et al.^8^, Naesstrom et al.^9^, and Raymaekers et al.^10^ were found to have serious risk of bias due to various reasons. In the studies by Chabardes et al.^4^ and Naesstrom et al.^9^, there was no sufficient analysis of confounding factors. Confounding factors include patient characteristics, which if omitted, leads to a lack of adjunct propensity score matching and sensitivity analyses and hence hinder the validation of the findings. The serious risk of bias in the studies by Holland et al.^5^ and Lopez-Sosa et al.^8^ stems from the potential bias in the selection of participants in those studies. In the study by Holland et al.^5^, a bias in the measurement of outcomes was noted as well. Bias in outcome measurement, alongside a deviation from the intended intervention, was also observed in the study by Li et al.^7^. The high risk of bias in Islam et al.^6^ and Raymaekers et al.^10^ is rooted in missing data in these studies. In the work of Islam et al.^6^, there was also a bias in the classification of interventions. Graat et al.^11^ was determined to have a critical risk of bias, due to the insufficient consideration of confounding factors, bias in the classification of intervention, bias due to deviations from intended interventions and bias in the measurement of outcomes.

Supplementary Figure 2**: Risk of bias analysis of all included randomised controlled trials (RoB-II tool).**


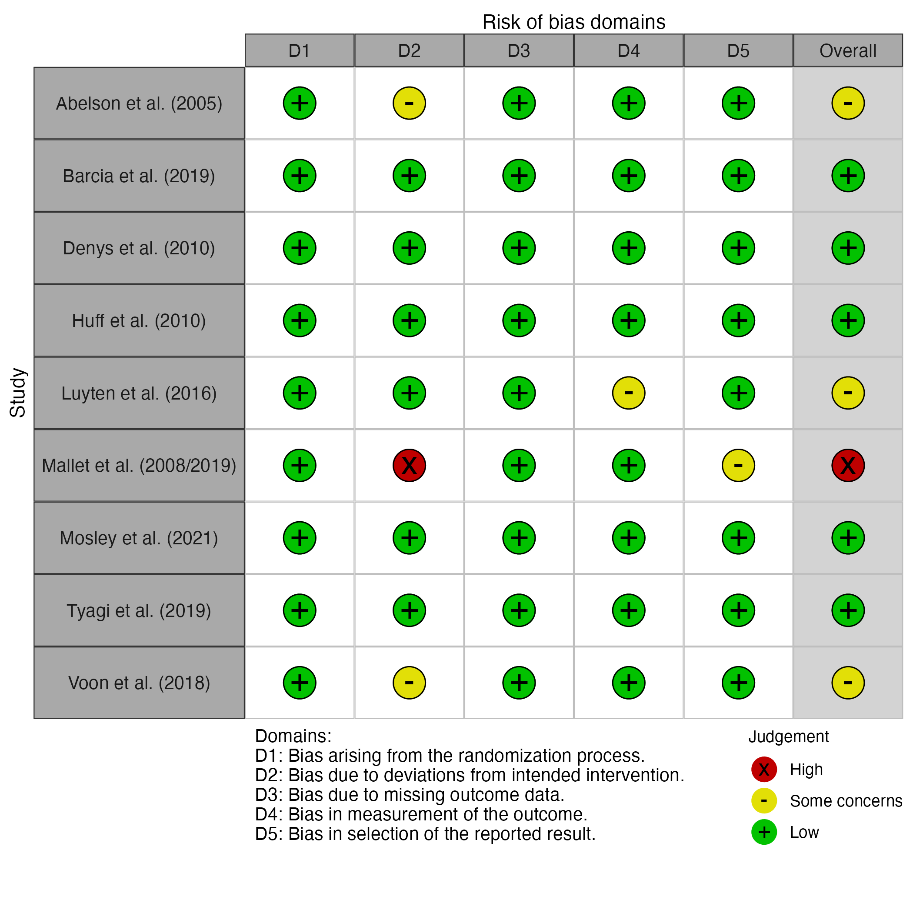


Supplementary Figure 2 shows the results of the risk of bias analysis of all included randomised studies using the RoB-2 tool^12^: Abelson et al.^13^, Voon et al.^14^ and Luyten et al.^15^ were judged to show some concern in terms of risk of bias, with Mallet et al. deemed to have a high levels of concern, as they show some bias in the measurement of outcomes or bias due to deviations from the intended intervention respectively.

Supplementary Figure 3: Forest plot displaying absolute change in Y-BOCS scores


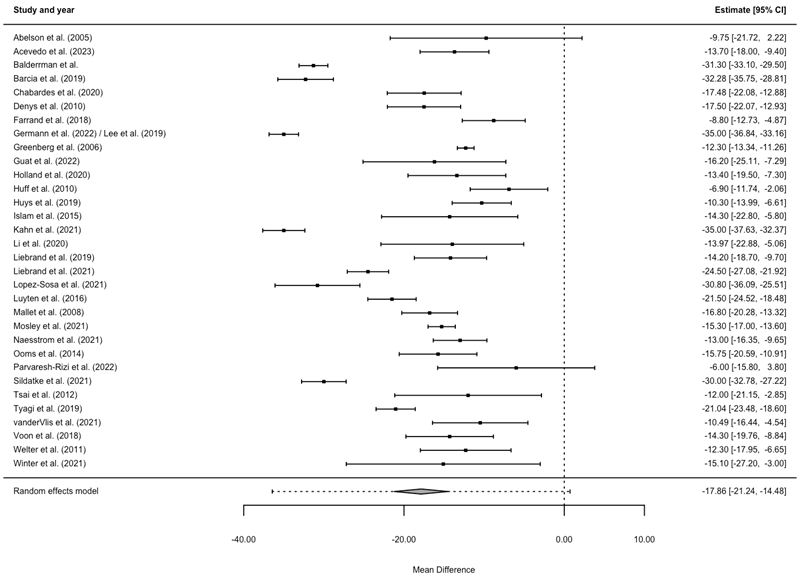


Supplementary Figure 3 shows a forest plot illustrating the absolute change in Y-BOCS scores, showing the mean difference with associated 95% CIs for each study. Missing standard deviations were derived from absolute change data using the calculation of the coefficient of variation for each study.

Supplementary Figure 4**: Forest plots displaying the mean change of the Y-BOCS according to study type.**
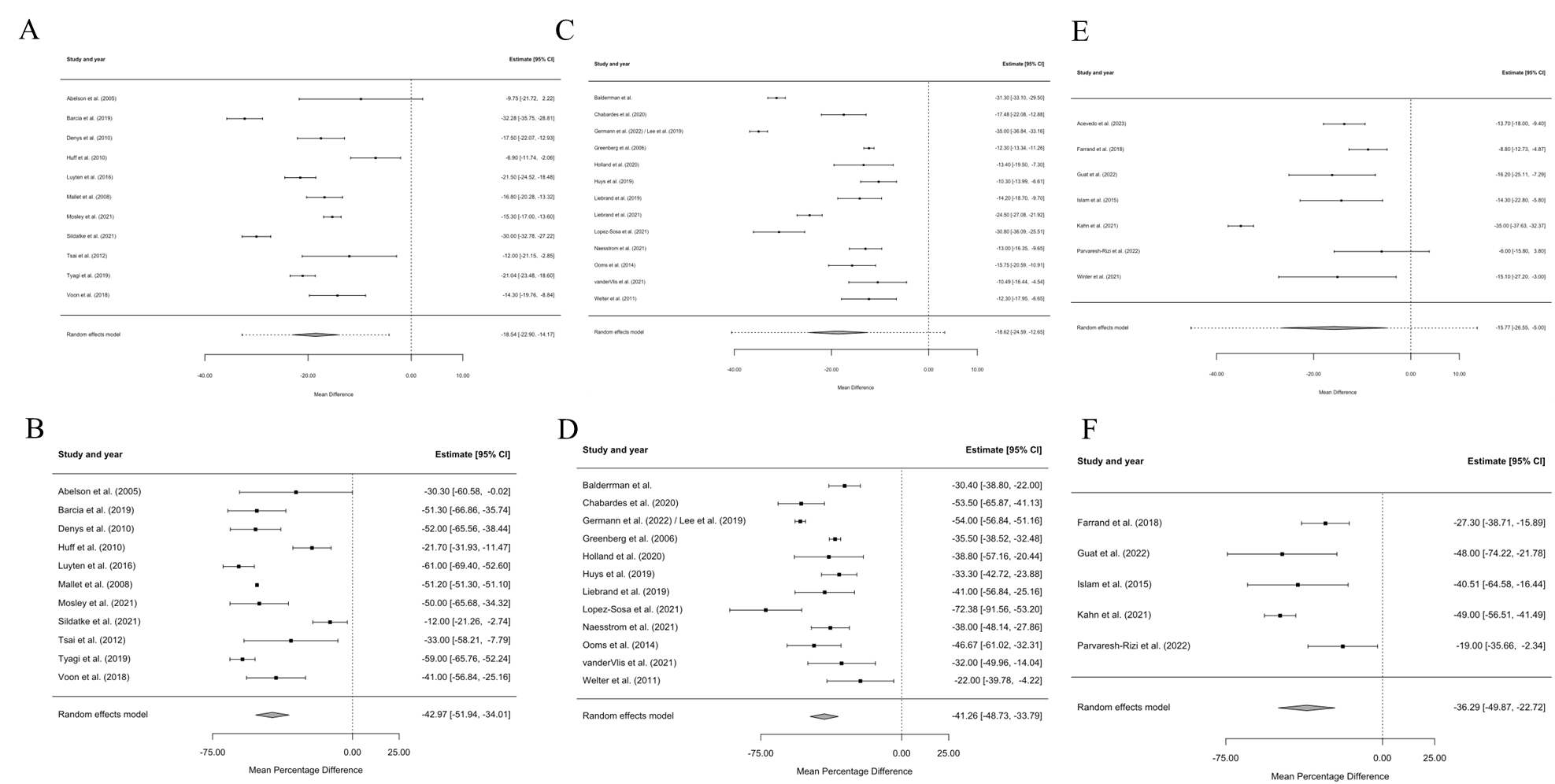


Supplementary Figure 4 shows forest plots displaying the effects of interventions on Y-BOCS (Yale-Brown Obsessive-Compulsive Scale) scores. Figures 4A, 4C and 4E present the absolute change in Y-BOCS scores across various studies, with the estimate and 95% confidence intervals (CIs) indicating the mean difference from the baseline. Figures 4B, 4D and 4F display the percentage change in Y-BOCS scores, again showing the mean difference with associated 95% CIs. The forest plots are grouped by study type with 4A and 4B comprising of randomised-controlled trials, 4C and 4D including cohort studies and 4E and 4F representing case-series. The plots include a summary estimate at the bottom, which represents the pooled effect size calculated using a random effects model. The dashed vertical lines denote the line of no effect, displaying the direction and magnitude of the intervention effects. The principal findings, summarized by the random effects model, indicate that DBS leads to a decrease in Y-BOCS score and, by extension, OCD disease severity, regardless of study design. Due to a lack of data availability, the mean difference for percentage change was missing for several studies and derivation from available statistics was not feasible as the required statistical assumptions were not met.

Supplementary Material 1**: A detailed account of the statistical methodology employed in this study.**

To ensure the reliability and robustness of the findings amidst the challenges of potential data discrepancies, a dataset was additionally constructed employing Predictive Mean Matching (PMM), being the primary chosen imputation method due to increased robustness given our missing-completely-at-random (MCAR) assumption regarding the original dataset. Chi-squared tests were applied to examine categorical clinical predictors across predefined response categories (responder, partial responder, non-responder). The statistical significance threshold was set at p < 0.05. Continuous clinical predictors were scrutinised through ANOVA tests, with those meeting the significance benchmark further subjected to Tukey's HSD post-hoc tests.

Univariate and stepwise multivariate regressions were performed on both the imputed (PMM-based) and non-imputed datasets. This dual approach was chosen to synthesise findings across different statistical models and to address the issue of missing data in the original dataset. To mitigate the potential for overfitting and to select the most relevant covariates for inclusion in the final multivariate model, a parameter boosting approach was employed, utilizing Ridge, LASSO, and Elastic Net regression techniques. Ridge regression adds a penalty equal to the square of the magnitude of coefficients to the loss function, thus shrinking the size of coefficients and helping to reduce model complexity. LASSO regression, on the other hand, adds a penalty equal to the absolute value of the magnitude of coefficients, which can lead to the exclusion of some variables entirely by reducing their coefficients to zero, thereby performing variable selection. Elastic Net regression is a hybrid method that includes both penalties of LASSO and Ridge, allowing for a balance between feature selection and multicollinearity mitigation. The utilisation of these regularisation techniques assists in identifying the most predictive covariates while also preventing the model from being too closely fitted to the particularities of the training data. The optimal set of covariates is selected based on their performance across these regularisation methods, ensuring that the final model incorporates variables that contribute to the robustness and generalisability of the predictions. These covariates were then considered alongside findings from the univariate and stepwise multivariate regression analyses of both the unimputed and PMM-imputed datasets, as well as systematic review findings and correlation analysis results. The combined approach, integrating regularisation methods with traditional regression analyses and evidence synthesis, allowed for the formulation of a comprehensive and informed multivariate regression model. Mixed-effect multivariate linear regressions and logistic regressions were then applied to continuous and categorical outcome variables, respectively. The final multivariate analyses were repeated without studies scoring high or critical risk of bias in our risk of bias assessment as part of our sensitivity analysis to ensure the robustness of our findings.

Continuous outcome variables were total Y-BOCS (short-term and long-term) and relative Y-BOCS change (baseline vs short-term or long-term), labelled as “Improvement”. While absolute scores provide insight into residual symptom severity, relative change accounts for variability in baseline severity and allows for a standardised comparison of treatment effects across studies. This dual approach ensures a more comprehensive assessment of response patterns and aligns with clinical decision-making, where both final symptom burden and magnitude of improvement are relevant considerations. Categorical outcome variables were classified as “Responder”, “Partial Responder”, and “Non-Responder”.

# References

1. Page MJ, McKenzie JE, Bossuyt PM, et al. The PRISMA 2020 statement: an updated guideline for reporting systematic reviews. *BMJ*. Mar 29 2021;372:n71. doi:10.1136/bmj.n71
2. Jeremy Howick, Iain Chalmers (James Lind Library), Paul Glasziou, et al. The Oxford Levels of Evidence 2. Oxford Centre for Evidence-Based Medicine - University of Oxford. Accessed October 24, 2023. <https://www.cebm.ox.ac.uk/resources/levels-of-evidence/ocebm-levels-of-evidence>
3. Sterne JA, Hernán MA, Reeves BC, et al. ROBINS-I: a tool for assessing risk of bias in non-randomised studies of interventions. BMJ. 2016;355. doi:10.1136/BMJ.I4919
4. Chabardes S, Krack P, Piallat B, et al. Deep brain stimulation of the subthalamic nucleus in obsessive-compulsives disorders: long-term follow-up of an open, prospective, observational cohort. J Neurol Neurosurg Psychiatry. Dec 2020;91(12):1349-1356. doi:10.1136/jnnp-2020-323421
5. Holland MT, Trapp NT, McCormick LM, et al. Deep Brain Stimulation for Obsessive-Compulsive Disorder: A Long Term Naturalistic Follow Up Study in a Single Institution. *Front Psychiatry*. 2020;11:55. doi:10.3389/fpsyt.2020.00055
6. Islam L, Franzini A, Messina G, Scarone S, Gambini O. Deep brain stimulation of the nucleus accumbens and bed nucleus of stria terminalis for obsessive-compulsive disorder: a case series. *World Neurosurg*. Apr 2015;83(4):657-63. doi:10.1016/j.wneu.20
7. Li N, Baldermann JC, Kibleur A, et al. A unified connectomic target for deep brain stimulation in obsessive-compulsive disorder. *Nat Commun*. 2020;11(1). doi:10.1038/S41467-020-16734-3
8. Lopez-Sosa F, Reneses B, Sanmartino F, et al. Nucleus Accumbens Stimulation Modulates Inhibitory Control by Right Prefrontal Cortex Activation in Obsessive-Compulsive Disorder. *Cereb Cortex*. Mar 31 2021;31(5):2742-2758. doi:10.1093/cercor/bhaa397
9. Naesstrom M, Hariz M, Stromsten L, Bodlund O, Blomstedt P. Deep Brain Stimulation in the Bed Nucleus of Stria Terminalis in Obsessive-Compulsive Disorder-1-Year Follow-up. *World Neurosurg*. May 2021;149:e794-e802. doi:10.1016/j.wneu.2021.01.097
10. Raymaekers S, Vansteelandt K, Luyten L, et al. Long-term electrical stimulation of bed nucleus of stria terminalis for obsessive-compulsive disorder. *Mol Psychiatry*. Jun 2017;22(6):931-934. doi:10.1038/mp.2016.124
11. Graat I, Balke S, Prinssen J, et al. Effectiveness and safety of deep brain stimulation for patients with refractory obsessive compulsive disorder and comorbid autism spectrum disorder; A case series. *J Affect Disord*. Feb 15 2022;299:492-497. doi:10.1016/j.jad.2021.12.089
12. Sterne JAC, Savović J, Page MJ, et al. RoB 2: a revised tool for assessing risk of bias in randomised trials. *BMJ*. 2019;366. doi:10.1136/BMJ.L4898
13. Abelson JL, Curtis GC, Sagher O, et al. Deep brain stimulation for refractory obsessive-compulsive disorder. *Biol Psychiatry*. Mar 1 2005;57(5):510-6. doi:10.1016/j.biopsych.2004.11.042
14. Voon V, Droux F, Chabardes S, et al. Dissociable Effects of Subthalamic Stimulation in Obsessive Compulsive Disorder on Risky Reward and Loss Prospects. *Neuroscience*. Jul 1 2018;382:105-114. doi:10.1016/j.neuroscience.2018.03.010
15. Luyten L, Hendrickx S, Raymaekers S, Gabriels L, Nuttin B. Electrical stimulation in the bed nucleus of the stria terminalis alleviates severe obsessive-compulsive disorder. *Mol Psychiatry*. Sep 2016;21(9):1272-80. doi:10.1038/mp.2015.124
